# *Fusarium oxysporum* f.sp. *ciceri* Race 1 Induced Redox State Alterations Are Coupled to Downstream Defense Signaling in Root Tissues of Chickpea (*Cicer arietinum* L.)

**DOI:** 10.1371/journal.pone.0073163

**Published:** 2013-09-13

**Authors:** Sumanti Gupta, Anirban Bhar, Moniya Chatterjee, Sampa Das

**Affiliations:** Division of Plant Biology, Bose Institute, Kolkata, West Bengal, India; Ghent University, Belgium

## Abstract

Reactive oxygen species are known to play pivotal roles in pathogen perception, recognition and downstream defense signaling. But, how these redox alarms coordinate *in planta* into a defensive network is still intangible. Present study illustrates the role of *Fusarium oxysporum* f.sp *ciceri* Race1 (Foc1) induced redox responsive transcripts in regulating downstream defense signaling in chickpea. Confocal microscopic studies highlighted pathogen invasion and colonization accompanied by tissue damage and deposition of callose degraded products at the xylem vessels of infected roots of chickpea plants. Such depositions led to the clogging of xylem vessels in compatible hosts while the resistant plants were devoid of such obstructions. Lipid peroxidation assays also indicated fungal induced membrane injury. Cell shrinkage and gradual nuclear adpression appeared as interesting features marking fungal ingress. Quantitative real time polymerase chain reaction exhibited differential expression patterns of redox regulators, cellular transporters and transcription factors during Foc1 progression. Network analysis showed redox regulators, cellular transporters and transcription factors to coordinate into a well orchestrated defensive network with sugars acting as internal signal modulators. Respiratory burst oxidase homologue, cationic peroxidase, vacuolar sorting receptor, polyol transporter, sucrose synthase, and zinc finger domain containing transcription factor appeared as key molecular candidates controlling important hubs of the defense network. Functional characterization of these hub controllers may prove to be promising in understanding chickpea–Foc1 interaction and developing the case study as a model for looking into the complexities of wilt diseases of other important crop legumes.

## Introduction

Plants are constantly threatened by various pathogens; however, disease manifestation seldom occurs [1]. Success of a pathogen largely depends on the irregularities of the well-coordinated host immune system [2]. Both plants and animals possessing ancient innate immunity share structural and strategic similarities [3]. In addition, animals have a specialized adaptive immune system that recruits mobile defender cells to sites of pathogen invasion, resulting in either pathogen death and/or arrest [4]. Unfortunately, plants lack this feature, and their sessile nature precludes escape from pathogens. Nevertheless, nature has equipped plants with highly specialized and orchestrated signal transduction machinery to compensate for the void of adaptive immunity [5]. Pathogen induced defense signals originating from an epicenter trigger multiple downstream effects, leading to the strengthening of the cell wall; site-specific trafficking of stored anticipins; *de novo* secretion of phytoalexins, small molecules, and secondary metabolites; generation of reactive oxygen and nitrogen species; and accumulation of phytohormones and pathogenesis related proteins. These features culminate in a hypersensitive response (HR)-mediated programmed cell death (PCD) at the infection site [6]. However, amidst all such signaling, the sequential events that lead to a well-coordinated defense network still appear to be blurred.

Plants use various molecular antennae and/or protein receptors to sense microbes [7]. Pathogen triggered immunity (PTI) and effector triggered immunity (ETI) are two modes of pathogen recognition utilized differently by the host [8]. The pathogen/microbe associated molecular patterns (PAMPs/MAMPs) are invariant epitopes of pathogen molecules such as flagellin, chitin, lipopolysaccharide etc that aid to virulence [9]. Often, pathogen induced host components, referred to as danger associated molecular patterns (DAMPs), such as callose, glucans, fructans etc also serve as elicitors [10]. These PAMPs/MAMPs/DAMPs are recognized by host pattern recognition receptors (PRRs), leading to the activation of PTI [11]. PTI, which is known to restrict a large number of pathogens induces a sudden imbalance in ionic concentrations, increases Ca^2+^ influx, activates cascades of mitogen-activated protein (MAP) kinases and protein phosphorylations, and induces altered hormone signaling [12]–[13]. However, a few pathogens successfully evade PTI and secrete effector proteins that contribute to effector triggered susceptibility (ETS) [2]. For counter defense, hosts secrete effector-specific R proteins, primarily nucleotide binding-leucine rich repeat (NB-LRR) domain containing “nibblers” that either interacts directly or through decoys/guardees with patho-effectors triggering a PTI like ETI [14]–[15]. ETI signaling events not only overlap with PTI but also compensate for its weakness [8]. HR mediated PCD and ethylene mediated systemic acquired resistance (SAR) are unique features of ETI. However, recent studies on *Arabidopsis* showed that MAMPs such as cellulose-binding elicitor lectin (CBEL), ethylene-inducing xylanase (EIX), and harpins induce SAR, whereas flg22 induces HR in plants, suggesting that the demarcation between PTI and ETI is more conceptual than factual [16]–[19]. Recent discoveries have indicated that quorum sensing in bacteria, siderophores of bacteria, and fungi serve as potential MAMPs and hydroxyproline containing glycopeptides (HypSys) and rapid alkalinization factors (RALFs) serve as DAMPs. However, their specific roles in defense are not well understood [10].

PTI and/or ETI mediated pathogen perception enkindles a repertoire of overlapping signals, of which generation of reactive oxygen species (ROS) and HR assisted PCD at the infection site appear to be the most important pathogenic event controlling resistance. In contrast, ROS generation adds to host phytotoxicity and hence is often found to be tightly regulated and detoxified through efficient scavenging systems in some resistant hosts. Such hosts are known to utilize the altered redox state of the infected cell to transmit downstream defense signals [20]. ROS, acting as primary signal inducers are also connected to Ca^2+^ sensors and protein phosphorylation networks through RBOH-NADPH (Respiratory burst oxidase homologue), oxidases, thioredoxins, peroxiredoxins, glutaredoxins, and/or NADPH. They are also known to induce secondary signal inducers, such as small peptides, hormones, lipids, cell wall fragments etc, cumulatively generating ROS waves that converge into the ROS network [21]. The role of ROS in fungal pathogenesis though less studied, but has recently put forth some obvious conclusions, showing that the ROS levels in the host are often controlled by the penetrating fungus itself [22]. Soluble sugars, sucrosyl oligosaccharides, and fructans have also been reported to contribute to oxidative stress regulation and ROS detoxification [23]. Besides, small molecule hormones, like salicylic acid (SA), jasmonic acid (JA), ethylene, abscissic acid (ABA), auxins, cytokinins, gibberellins (GA), and brassinosteroids, are also considered as central players [24]. Signaling pathways involving these hormones cross communicate either in an antagonistic and/or synergistic manner, providing a fine tune balance to the host.

Among several well discussed pathosystems, detailed molecular dynamics of plant-fungal interactions are undoubtedly scarce. Moreover, reports on legume–fungus antagonism are either less or if present, have failed to provide insights on the molecular interactomics; lack of functional annotations probably accounting for such failures. Additionally, a low degree of resemblance between model and crop legumes according to genome conservation analyses across model versus crop legumes suggests the need for studying crop–pathogen interactions individually, rather than generalizing the immune responses based on model species [25]. The present study describes the molecular details of innate immune responses in chickpea induced after Foc1 invasion. Chickpea which ranks third in the world list of important legumes and first in the Indian list is known to meet with 10–15% annual losses due to vascular wilt caused by the Foc1.

This seed and/or soil borne pathogen is known to enter the host through breaches between the tap root and its branches and colonize the xylem vessels of the root–shoot junction [26]. The pathogen comprises of two pathotypes i.e the yellowing type and the wilt causing type, of which the latter is known to cause severe devastations under favorable environmental conditions [26]. Amongst several wilt causing races of *Fusarium oxysporum* f. sp. *ciceri*, Race1 is known to have a wide geographical distribution, resulting in a large amount of crop loss. Although wilt is primarily managed by breeding programs, the variability and mutability of pathogenic races lead to the breakdown of natural resistance. However, a lack of information regarding resistant gene/genes indicates that this field is open for future research. Details of *in planta* pathogen progression and colonization events of Foc1have already been reported by our group and others [27]–[29]. Previous results highlighted some special features in chickpea–*Fusarium* case study in which vascular clogging and resultant HR was linked to susceptibility rather than to the classical resistance. However, it should be emphasized that resistance and PCD do not necessarily correlate [30]–[31].

Previous reports had suggested that wound inducing Foc1 was sensed early by the host. Besides, this host–pathogen interplay triggered transcriptomic reprogramming directed towards regulating primary host metabolism, where sugar molecules served as defense signal modulators [32]. However, recent updates in the chickpea EST database related to the chickpea–*Fusarium* case study have not only contributed significantly to the understanding of molecular dynamics, but have also necessitated further studies examining the details of this interaction [29]. Moreover, the knowledge of the sequential association of key nodal and internodal molecular candidates that ultimately interconnect to form a coordinated defense-signaling network with PTI and ETI signal overlaps is still elusive and thus was the focus of this study. Earlier reports had suggested wound mediated pathogen invasion and HR at the site of infection [32]. Such wounding responses are known to generate ROS and change the redox state of the infected host [20]. The present study illustrates the mode of pathogen entry and investigates the expression pattern of several redox responsive transcripts such as ROS generators and scavengers, cytochrome-dependent redox signal transducers, and intracellular ROS signal transducers during pathogen progression and establishment. These defense signals generated by ROS molecules are known to transmit downstream signals to the host interior with the help of cellular transporters. The present study also demonstrates how these altered redox signals are transmitted to host interior through several intracellular transporters. Moreover, defense networks are tightly regulated by several transcription factors (TFs) that modulate the downstream expression of defensive genes either directly or by influencing the expression of their associates. The present study explains the probable role of several transcription factors in regulating the chickpea–*Fusarium* defense network. Previous reports had emphasized the role of sugars as signaling molecules [27], [32]. In the present study, the role of sugar metabolizers as intracellular signal transmitters and modulators are also examined.

## Materials and Methods

### Plant and fungal materials

Experiments were performed with wilt susceptible (JG62) and wilt resistant (WR315) chickpea (*Cicer arietinum* L.) seeds obtained from International Crops Research Institute for Semi-Arid Tropics (ICRISAT), Patancheru, Andhra Pradesh, India. Seeds were sown in mixture of synthetic soil and sand (1∶1) and plantlets were maintained under optimum greenhouse conditions at temperatures ranging from 22–28°C, 35–40% humidity, and 16∶8 hours photoperiod of day and light, respectively, as described by Haware & Nene [26].

Fungal strain of *Fusarium oxysporum* f. sp. *ciceri* (Foc1), obtained from ICRISAT, was purified and maintained according to Summerell, Salley & Leslie [33]. Spores were harvested and stored at −80°C in 30% glycerol until further use.

### Fungal infection assay

Sterilized seeds of both JG62 and WR315 were germinated in sterile synthetic soil. Two week old seedlings were used for the assay. Infection was induced by sick soil method as described by Gupta *et al.* [27]. Optimum growth conditions were offered to both control and experimental sets. Roots of infected and control plants were collected on the following days post inoculation (dpi): i.e. 1dpi, 1.5dpi, 2dpi, 3dpi, 4dpi, 7dpi and 12dpi. Root samples were weighed into 1 g aliquots, flash frozen in liquid nitrogen, and stored at −80°C for further analysis.

### Confocal scanning laser microscopy (CSLM)

Root samples of both infected and uninfected JG62 and WR315 plants were collected at 4dpi, 7dpi, and 12dpi, immediately incubated in a mixture of 75% ethanol and 15% acetone for 15 minutes, and finally fixed with 50% methanol and 10% acetic acid. Serial sectioning of root samples were performed and approximately 50 μm thick sections were selected for microscopy.

Selected sections were stained individually with trypan blue, aniline blue (Himedia), propidium iodide, and SYTOX green (Invitrogen) as well as with combinatorial stain containing both trypan blue-aniline blue and propidium iodide-SYTOX green following the protocol suggested by Bhadauria *et al*. [34] and Truernit & Haseloff [35]. Following staining, all the sections were thoroughly washed and mounted on grease-free glass slides using Keiser's gelatin (Merck). Imaging was performed using CLSM Model LSM-510 Meta (Carl Zeiss) with excitation and emission wavelengths as mentioned in Table S1. All images were analyzed using LSM-510 software. Three biological replicates were used for all microscopic studies.

### Lipid peroxidation assay

#### a) Biochemical assay

Biochemical assay was carried out using enzyme extracts isolated from infected (2dpi, 3dpi, 4dpi, 7dpi, and 12dpi) and uninfected roots. Degree of lipid peroxidation in roots was measured using 2-thiobarbituric acid (TBA) assay [36]. 800 μL of TBA reagent, containing 15% w/v TBA in 0.25 N HCl was mixed with 400 μL of enzyme extract and heated in a 100°C water bath for 15 minutes. The mixture was then cooled and centrifuged at 1000×*g* for 10 minutes. The supernatant was removed and absorbance measured using a Beckman and Coulter DU-520 UV/VIS spectrophotometer at 535 nm. Biological and technical replications were performed in triplicates and error calculated (Table S2).

#### b) Immunoblot Experiment

Total protein was extracted from infected (2dpi, 4dpi, 7dpi, and 12dpi) and uninfected roots of JG62 and WR315 plants using the protocol summarized by Wang *et al*. [37]. Extracted proteins were used for immunoblot experiments. Lipid peroxidation was reconfirmed by immunoblot experiments using OxiSelect Malondialdehyde (MDA) Immunoblot Kit (Cell biolabs) [38]–[39]. Total protein (10 µg) from uninoculated and inoculated roots of 2dpi, 4dpi, 7dpi, and 12dpi were used for immunoblotting. Protein was run on 10% SDS-PAGE and blotted according to the manufacturer's instructions. Three biological replicates were used for the assay.

### RNA and cDNA preparation

Roots of uninfected and infected plants of both varieties were collected at 1dpi, 1.5dpi, 2dpi, 3dpi, 4dpi, and 7dpi and frozen in liquid nitrogen. Total RNA was isolated using TRI reagent kit (Sigma-Aldrich) according to the manufacturer's instructions. First strand cDNA was synthesized using Revert Aid First Strand cDNA Synthesis Kit (Fermentas) following the manufacturer's guidelines, checked on 1.1% agarose-Tris acetate EDTA (TAE, pH 8.0) gel (Merck), and quantified in NanoDrop spectrophotometer (ND-1000, NanoDrop Technologies) at 280 nm. cDNAs were stored at −80°C until further use.

### Quantitative real-time PCR (qRT-PCR)

Quantitative transcriptome profiling of β-1,3-endo glucanase, several redox related transcripts, cellular transporters, TFs, and sugar metabolizers was conducted for infected (1dpi, 1.5dpi, 2dpi, 3dpi, 4dpi, and 7dpi) and uninfected plants of both varieties (Table 1, Table S3). qRT-PCR was performed on Bio-Rad iCycler (Bio Rad iQ5) with SyBr green. Reaction mix (20 µL) containing SyBr green qPCR Super Mix (2×) (Bio Rad), 25 ng cDNA, and 0.3 μM of forward and reverse primers was taken for PCR (Table S4). The following cycle conditions were used: 95°C for 5 minutes, followed by 40 cycles at 95°C for 30 seconds, 50–55°C for 30 seconds, and 72°C for 30 seconds [32]. Melt curve was analyzed to evaluate primer specificity. Glyceraldehyde-3-phosphate dehydrogenase (GAPDH) was used as the internal control [40]. The mean fold change of all the genes was calculated following 2^−δδCt^ method [41]. All experiments were conducted in triplicates. Standard deviation and standard error was calculated for each transcript. Unpaired student's t test was performed and p value (<0.05) calculated for entire data set (Table S5, Table S6).

**Table 1:** List of transcripts, their abbreviations and their homologies used for qRT-PCR and pathway construction.

EST names	Abbreviations used in the study	Abbreviated gene names used in pathway generation	Chickpea Transcriptome Database (CTDB) homologues	TAIR homologues
**1. REDOX RESPONSIVE TRANSCRIPTS**				
** A. ROS GENERATORS & SCAVENGERS**				
Respiratory burst oxidase homologue	RBOH	ATRBOH_F	TC16083	AT1G64060
Peroxidase	-	-	TC04331	AT2G38380
Cationic peroxidase	-	OCP3	TC18397	AT5G11270
Iron superoxide dismutase	(Fe-SOD)	FSD1	TC17160	AT4G25100
Glutathione S transferase (TAU 26)	GST-(TAU-26)	-	TC01997	AT1G17190
** B. CYTOCHROME DEPENDENT REDOX SIGNAL TRANSDUCERS**				
Cytochrome b561 Fe reductase	FRO	FRO7	TC15027	AT5G49740
FAD linked oxidase family protein	-	-	TC16285	AT4G36400
NADH cytochrome b5 reductase	CBR	ATCBR	TC14723	AT5G17770
** C. INTRACELLULAR ROS SIGNAL TRANSDUCERS**				
NADP dependent oxidoreductase	-	-	TC15458	AT5G16990
Quinone oxidoreductase	FQR	FQR1	TC18358	AT5G54500
Fe (II) oxidoreductase	-	-	TC17646	AT5G24530
F-type thioredoxin	TRX	TRX3	TC18037	AT5G42980
H+ transporting ATPase	-	-	TC14586	AT3G28710
**2. CELLULAR TRANSPORT RELATED TRANSCRIPTS**				
** A. INTRACELLULAR TRANSPORTERS**				
ABC transporter like protein	-	-	TC05017	AT2G34250
Substrate transporter (carbohydrate)	-	-	TC16724	AT1G54730
Heavy metal transporter/ detoxyfying protein (FAR1 related sequence 6: FRS6)	-	-	TC07483	AT1G52520
Translocase (chloroplast 34)	-	-	TC14372	AT5G05000
Polyol transporter protein	PLT	PLT5	TC04362	AT3G18830
** B. CELLULAR TRAFFICKINGTRANSPORTERS**				
Vacuolar sorting receptor	VSR	VSR1	TC10023	AT3G52850
Clathrin coat assembly protein	-	-	TC17520	AT4G24550
Secretory carrier membrane protein	-	-	TC15013	AT2G20840
Nuclear pore complex protein	-	-	TC01108	AT5G51200
Intrinsic protein of tonoplast	TIP	TIP2	TC07483	AT3G26520
** C. INTRACELLULAR TRANSPORTATIONSIGNAL GENERATORS**	-	-	TC04434	AT4G00630
TRK(A-N) signaling factor	-	-	-	-
Type II B calcium ATPase	ACA	ACA2	TC01484	AT4G37640
**3. TRANSCRIPTION FACTOR RELATED TRANSCRIPTS**				
** A. TRANSCRIPTION FACTOR CONTAINING BASIC DOMAINS**				
bZIP domain containing protein	-	-	TC16286	AT5G42910
Homoeodomain leucine zipper like protein	REV	REV	TC05808	AT5G60690
MYB like transcription factor	MYB	MYB106	TC09491	AT3G01140
Helix loop helix domain containing transcription factor	-	-	TC15129	AT1G05805
Zinc finger (CCHC type)	AZF	AZF2	TC00757	AT3G19580
Heat shock family protein	HSF	HSF3	TC04961	AT5G16820
** B. TRANSCRIPTION FACTOR ASSOCIATORS**				
Polynucleotidyl transferase (FAR1)	-	-	TC34562	AT4G12850
Initiation factor 4a	-	-	TC02836	AT3G13920
Prefoldin (ILR3)	-	-	TC17935	AT5G54680
High mobility group B like protein	HMG	HMGB3	TC07452	AT1G20696
**4. SUGAR METABOLISM RELATED GENES**				
Sucrose synthase	SUS	SUS4	TC11959	AT3G43190
β Amylase	BAM	BAM1	TC06159	AT3G23920
Invertase	-	-	TC05218	AT3G05820

### Sugar assay

Soluble sugar was extracted from the roots of induced and uninduced JG62 and WR315 plants at 1dpi, 1.5dpi, 2dpi, 3dpi, 4dpi, 5dpi, and 7dpi. Roots were immediately oven dried at 68°C for 2–3 days, crushed in mortar and pestle, and distributed in 50 mg aliquots. During extraction, freshly prepared 80% methanol was added to powdered roots, mixed by vortexing, incubated for 2 hours at room temperature, and centrifuged at 8000 rpm for 10 minutes. The supernatant was transferred to a sterile microcentrifuge tube, vacuum dried, and dissolved in 100 μL of HPLC grade water (Merck) [42]. Sugar contents were measured using the Sucrose/d-Glucose/d-Fructose Assay Kit (R-Biopharm) according to the manufacturer's instructions. Enzymatic conversion of sugar molecules (Glucose and Sucrose) producing equimolar amount of NADPH was detected by spectrophotometer (Beckman-Coulter, DU-520) at 340 nm. Sugar concentration was expressed as micrograms of sugar per milligram root dry weight. Experiments were conducted in triplicates and errors were calculated (Table S7).

### Network analysis

Several redox-responsive transcripts, cellular transporters, TFs, and sugar-metabolizing ESTs identified in the chickpea–*Fusarium* case study [27], [29] were subjected to BLAST analyses, their *Arabidopsis* homologous genes identified and used as inputs for network generation using Pathway Studio Software (version7.1) (Ariadne Genomics, Table 1, Table S3). The interrelationship between redox-related transcripts, cellular transporters, TFs, sugar metabolizing components, and their regulators were analyzed by Pathway Studio Software using the neighbor joining method; samples with a degree of correlation of 1, i.e only neighbors having direct relationship to the gene/gene products, were used as inputs.

## Results

### Foc1 invasion and colonization induces callose degradation, membrane disruption, and tissue damage

**Figure 1:**
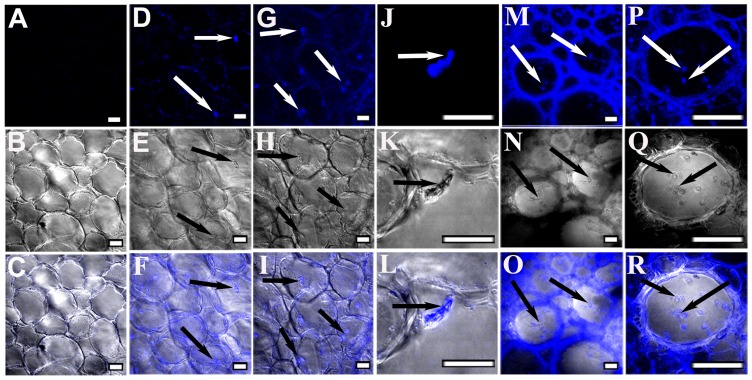
Confocal scanning laser microscopic images showing Foc1 progression and colonization in roots of JG62 plants. (A–C) corresponds to root sections of uninduced plants. (D–F) represent root sections of JG62 plants after 4dpi; (G–I) after 7dpi; (J–L) after 7dpi (magnified view); (M–O) after 12dpi; (P–R) after 12dpi (magnified view). (A, D, G, J, M, P) represents fluorescent images. (B, E, H, K, N, Q) represents differential interference contrast (DIC) images. (C, F, I, L, O, R) represents merged images. Bars represent 20 µm.

**Figure 2:**
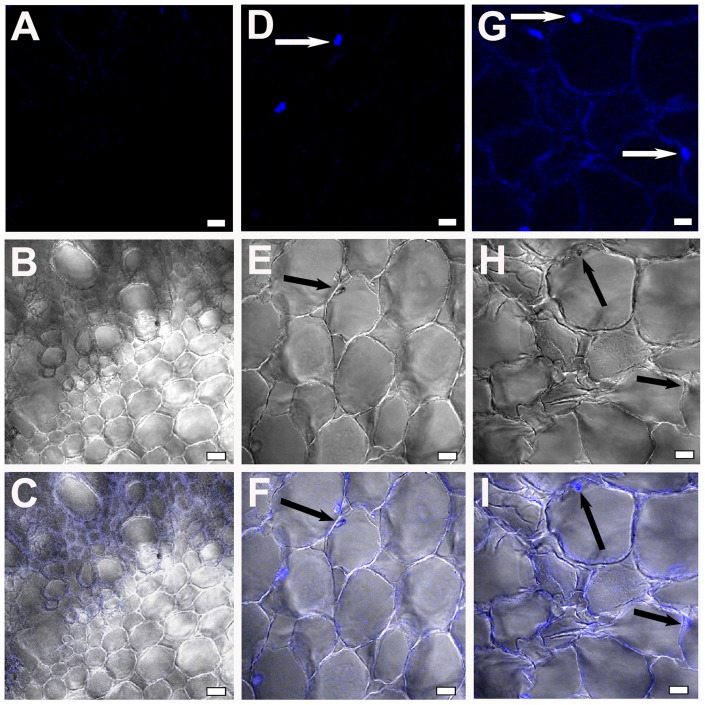
Confocal scanning laser microscopic images showing Foc1 progression and colonization in roots of WR315plants. (A–C) correspond to root sections of uninduced plants. (D–F) represent root sections of WR315 after 7dpi; (G–I) showing root sections after 12dpi. A, D, G represent fluorescent images; B, E, H represent DIC images and C, F, I represent merged images. Bars represent 20 µm.

Pathogenic events such as callose degradation, membrane disruption, and tissue damage were monitored using CSLM. Trypan blue, which can bind cell wall components, was used to examine fungal progression [34]. Root sections of uninduced JG62 and resistant WR315 plants served as controls (Fig. 1A–C, Fig. 2A–C). In compatible interaction, fungal pegs were initially observed in cortical root cells at 4dpi (Fig. 1D–F) which extensified at 7dpi (Fig. 1G–I). Magnified images of 7dpi showed attempted penetration of an infecting hypha arising from the cell cytosol, which pierced the adjoining cell (Fig. 1J–L). Ramification and colonization further intensified at 12dpi with visible mycelial mesh within the xylem vessels and adjoining tissue (Fig. 1M–O). The presence of microconidia both singly and in clusters inside the xylem vessel indicated stable establishment of the fungus within the susceptible host (Fig. 1P–R). Gradual loss of tissue integrity was noticed. However, in the incompatible interaction, the resistant host WR315 showed contrasting features. Although, infection pegs were visible at 7dpi (Fig. 2D–F), but showed noticeably less intensification even at 12dpi (Fig. 2G–I).

**Figure 3:**
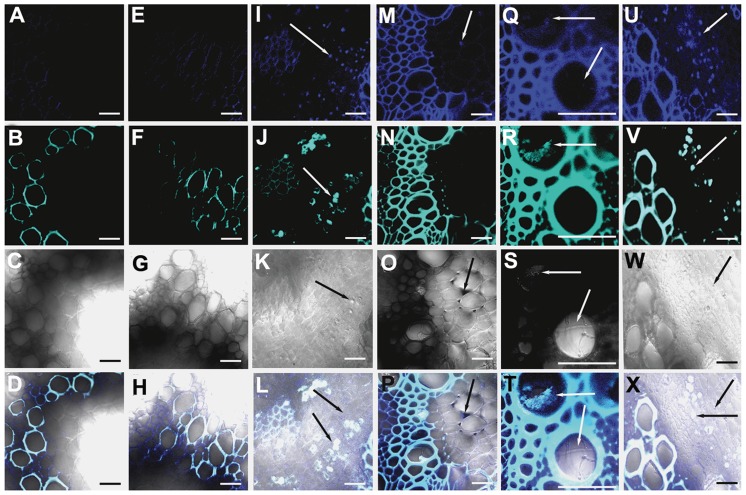
Confocal scanning laser microscopic images highlighting Foc1 ramification and callose degradation products in chickpea roots. (A–D) and (E–H) represents root sections of uninduced JG62 and WR315 plants respectively. (I–L) and (Q–T) corresponds to root sections of infected JG62 at 4dpi and 12dpi. (M–P) and (U–X) corresponds to infected root sections of infected WR315 at 4dpi and 12dpi. A, E, I, M, Q, U represent fluorescent images stained with trypan blue; B, F, J, N, R, V represent fluorescent images stained with aniline blue; C, G, K, O, S, W represent DIC images; D, H, L, P, T, X represent merged images. Bars correspond to 20 µm.

**Figure 4:**
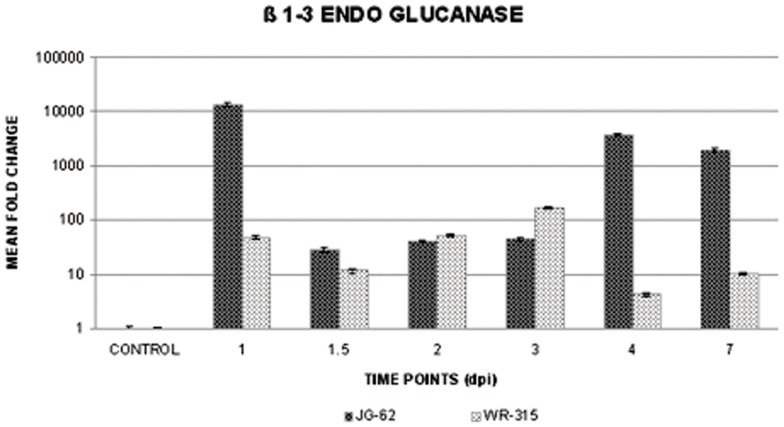
qRT-PCR showing expression of beta 1,3 endo glucanase in chickpea roots. Relative expression of beta 1,3 endo glucanase in susceptible JG62 and resistant WR315 roots of chickpea plants in response to Foc1 infection. Bars represent the standard errors (n = 3).

Trypan blue and aniline blue combinatorial stain was used to analyze pathogen progression and callose degradation due to Foc1 incursion. Uninduced root sections of both varieties were used as controls (Fig. 3A–H). Onset of callose degradation was marked in fungal infested cortical root cells in JG62 plants at 4dpi (Fig. 3I–L). At 12dpi, formation of mycelial network and clogging of xylem vessels with callose degraded products was noticed in susceptible plants (Fig. 3Q–T). In resistant plants, fungal pegs were observed at 4dpi, but callose degradation was absent (Fig. 3M–P). However, at 12dpi, minimal amounts of callose degradation product at root cortical cells were observed. But, the vessels remained unclogged (Fig. 3U–X). qRT-PCR of β-1,3-endo glucanase showed enhanced expression in the susceptible variety compared to resistant plants at 1dpi, 4dpi, and 7dpi. However, 2dpi showed comparable expression in both the infected varieties, while 3dpi exhibited a reversed expression pattern in resistant plants (Fig. 4).

Propidium iodide and SYTOX green effectively stain cell wall and nuclei of membrane-compromised cells [35]. However, in the present study, root sections of uninduced plants subjected to dual staining with dyes showed fluorescent signals in both the membrane and the nuclei (Fig. S1. A–H). Tissue sectioning, fixation, and dehydration likely led to membrane injury resulting in abnormal stain uptake even by uninduced samples. Infected root sections of JG62 and WR315 plants also showed similar staining properties (Fig. S1. I–L, M–P). However, membrane integrity was disrupted in JG62 plants at 12dpi (Fig. S1. I–L), whereas WR315 plants showed uniform cell layers as uninfected controls (Fig. S1. M–P).

**Figure 5:**
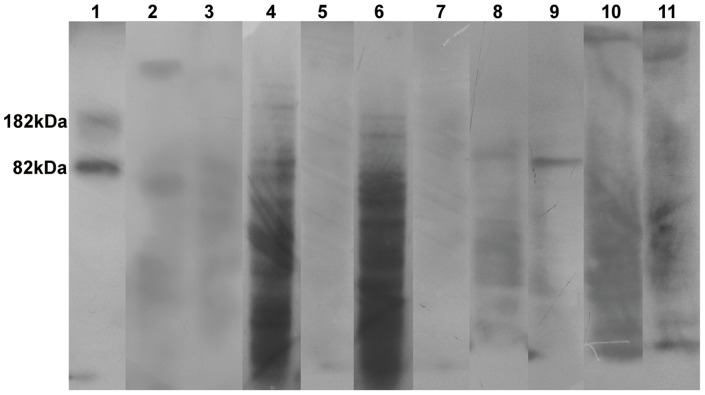
Immunoblot assay showing lipid peroxidation mediated membrane damage. Lane1; positive control showing MDA-BSA bands at 82kDa and 182kDa respectively. Lane 2 and 3; total protein profile of uninduced roots of JG62 and WR315 respectively. Lane 4, 6, 8 and 10; total protein profile of infected roots of JG62 at 2dpi, 4dpi, 7dpi and 12dpi respectively. Lane 5, 7, 9 and 11; total protein profile of infected roots of WR315 at 2dpi, 4dpi, 7dpi and 12dpi. Each lane contains 10 µg of total protein uniformly.

Pathogen induced membrane damage was confirmed using biochemical and immunoblot assays to observe lipid peroxidation-mediated membrane injury. Biochemical assay was performed using TBA as a substrate that is known to form conjugates with MDA. These MDA byproducts are formed through oxidative reactions involving reactive superoxide molecules and polyunsaturated fatty acids present in the membrane lipid bilayer. Lipid peroxidation, an indicator of membrane damage, was found to be high in JG62 plants at 2dpi, 4dpi, and 7dpi than in uninduced control plants, indicating pathogen ingress and colonization. However, samples at 12dpi showed the lowest peroxidation levels. Lipid peroxidation levels showed an initial increase at 4dpi in WR315 plants as compared to uninduced control plants, but the levels decreased at later time points (Fig. S2). However, since TBA can form conjugates with other non-MDA molecules, non-MDA–TBA byproducts often mask the absorption of MDA–TBA products during spectrophotometric detection. Hence, immunoblot experiments were conducted in which only MDA–protein adducts were detected. Immunoblot experiments conducted with anti-MDA antibody showed increased lipid peroxidation in JG62 plants from 2dpi to 12dpi, concurrent with pathogen progression. However, an abrupt decrease was observed at 7dpi (Fig. 5). In WR315 plants, peroxidation gradually increased from 7dpi onwards (Fig. 5). Biochemical and immunoblot assays showed dissimilar results, probably due to different substrate identities for MDA–TBA and MDA–protein conjugates in biochemical and immunoblot experiments, respectively. Thus, only the results obtained through immunoblot experiments were considered to be better explanatory in later section of the study.

### Fungal attack induces nuclear adpression and cell shrinkage

**Figure 6:**
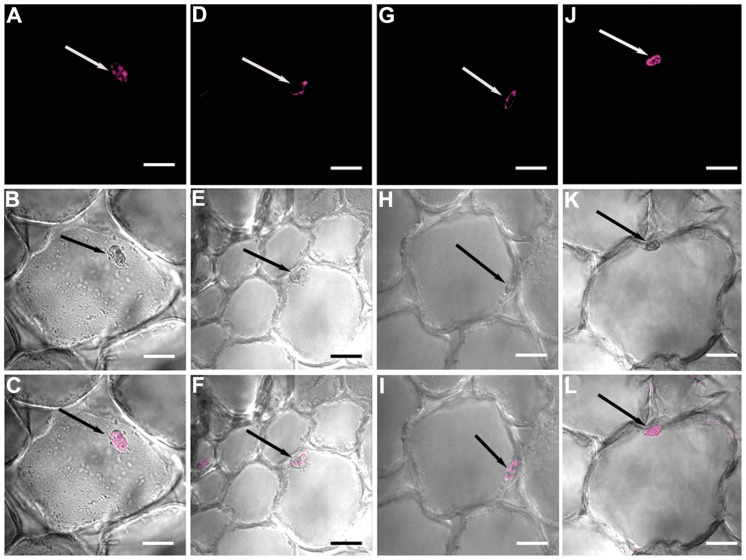
Confocal microscopic images showing Foc1 infection induced nuclear migration in root cells of chickpea plants. (A–C) correspond to root sections of uninduced JG 62 plant; (D–F) correspond to root sections of infected JG62 plant at 7dpi; (G–I) of infected JG62 plant at 12dpi; (J–L) correspond to root sections of infected WR315 plant at 12dpi. A, D, G, J represent fluorescent images; B, E, H, K represent DIC images; C, F, I, L represent merged images. Bars correspond to 10 µm.

Propidium iodide is known to stain the cell wall and nuclei of membrane-compromised cells and was found to exceptionally stain uninduced root samples of JG62 plants in the present study (Fig. 6A–C). However, this experiment led to an interesting revelation of altered cell size and nuclear position of Foc1 infected cells. In uninduced root cells, the cell size and nuclear position appeared to be normal (Fig. S3A), whereas in infected JG62 cells, cell size was altered and nuclei were gradually adpressed to the cell membrane at 7dpi (Fig. 6D–F, Fig. S3B). Cell shrinkage and nuclear adpression became more prominent at 12dpi (Fig. 6G–I, Fig. S3C). In WR315, cell shrinkage and nuclear adpression were evident at 12dpi, but to a lesser extent than in infected JG62 plants (Fig. 6J–L, Fig. S3D).

### Foc1 entry causes expressional alterations of redox responsive transcripts

**Figure 7:**
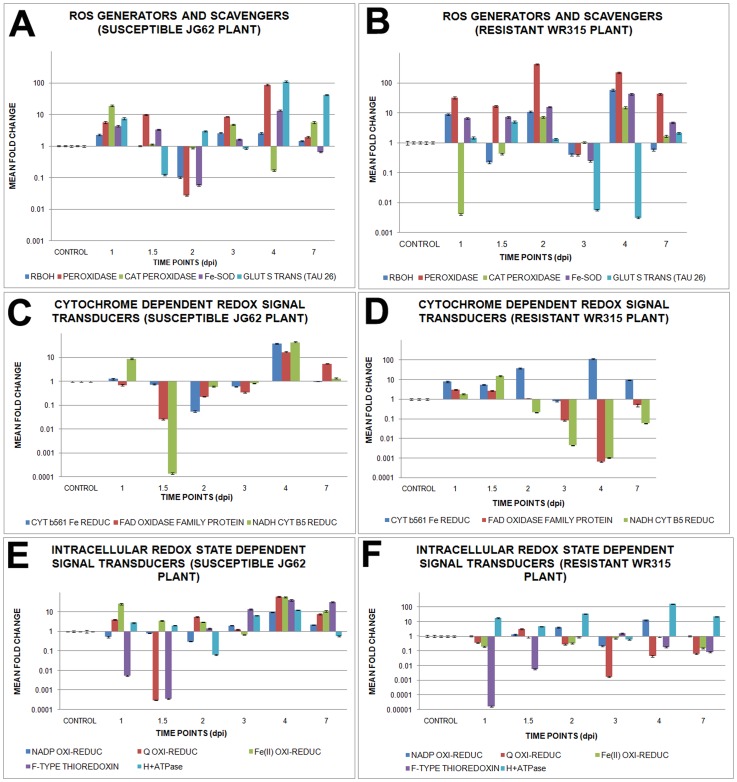
Relative expression of redox signalling genes in chickpea roots in response to Foc1 induction. (A) and (B) represents relative expression profile of ROS generators and scavengers like respiratory burst oxidase homologue (RBOH), peroxidase, cationic peroxidase, iron superoxide dismutase (Fe-SOD) and glutathione s transferase TAU26 (GST-TAU26) in uninduced and induced JG62 and WR315 roots respectively; (C) and (D) represents relative expression pattern of cytochrome dependent redox signal transducers like cytochrome b561 Fe reductase, FAD linked oxidase family protein and NADH cytochrome b5 reductase in uninduced and induced JG62 and WR315 roots respectively; (E) and (F) represents relative expression profile of intracellular ROS signal transducers like NADP oxido-reductase, quinone oxido-reductase, Fe(II) oxido-reductase, F type thioredoxin and H^+^ transporting ATPase in uninduced and induced JG62 and WR315 roots respectively. Bars represent the standard errors (n = 3).

Expressional profiling of ROS generator such as respiratory burst oxidase homologue showed comparatively less expression in susceptible JG62 plants, while resistant WR315 plants showed expressional fluctuations throughout pathogen progression. Peroxidase expression was increased by several-fold in WR315 plants compared to JG62 plants, with a sharp expressional drop at 3dpi. In contrast, cationic peroxidase was increased in JG62 plants, while WR315 plants showed low expression throughout the study period, except at 2dpi, on which cationic peroxidase expression increased marginally. However, the expression increased sharply at 4dpi. Iron superoxide dismutase (Fe-SOD) expression was higher in WR315 plants on all days except 3dpi, on which it showed significantly decreased expression. Glutathione S transferase (TAU26) (GST-TAU26) showed a sequential rise and drop in expression in JG62 plants while WR315 plants maintained overall low expression except at 1.5dpi, which showed highest expression (Fig. 7A and B, Fig. S4).

Expression of cytochrome dependent redox signal transducers when compared showed uniform upregulation of cytochrome b561 ferric reductase in WR315 plants, with both plants showing comparable expression only at 3dpi; however, in case of FAD linked oxidase family protein expression increased at 4dpi and 7dpi in JG62 plants. NADH cytochrome b5 reductase showed an overall low expression in both plants, with only JG62 plants showing an expressional hike at 1dpi and 4dpi, while this hike was recorded at 1.5dpi in WR315 plants (Fi.7C and D, Fig. S4).

Additionally, several intracellular ROS signal transducers exhibited differential profiling during pathogen progression. Except for 3dpi and 7dpi, NADP oxidoreductase showed comparatively enhanced expression in WR315 plants, while quinone oxidoreductase showed the reverse pattern throughout pathogen progression. Both Fe (II) oxidoreductase and F-type thioredoxin showed uniformly low expression in WR315 plants compared to JG62 plants. H+ ATPase showed upregulation throughout the study in WR315 plants, with only 3dpi showing a sharp downregulation in expression (Fig. 7E and F, S4).

Amongst all the redox related transcripts seven, such as redox regulatory respiratory burst oxidase homologue F (RBOHF) (respiratory burst oxidase homologue), thioredoxin 3 (TRX3) (homologue of F-type thioredoxin), overexpressor of cationic peroxidase 3 (OCP3), (homologue of cationic peroxidase), flavodoxin-like quinone reductase 1 (FQR1) (homologue of quinone oxidoreductase), iron superoxide dismutase 1 (FSD1) (homologue of Fe-SOD), NADH cytochrome b5 reductase (CBR), and Fe (II) oxidoreductase 7 (FRO7) (homologue of Fe (II) oxidoreductase) showed interactions in the pathway. RBOH, indicated as a positive regulator of ROS and hypersensitive response, showed linked with TRX3 and OCP3, with hydrogen peroxide and abscisic acid acting as intermediate small molecules. FQR1, FSD1, CBR, and FRO7 formed independent nodes. OCP3 appeared as negative regulator of infection response, while FRO7 positively regulated iron homeostasis and photosynthesis. Cell fate and oxidative stress were found to be regulated by TRX3 (Fig. S5).

### Cellular transporters display differential expressional profiles

**Figure 8:**
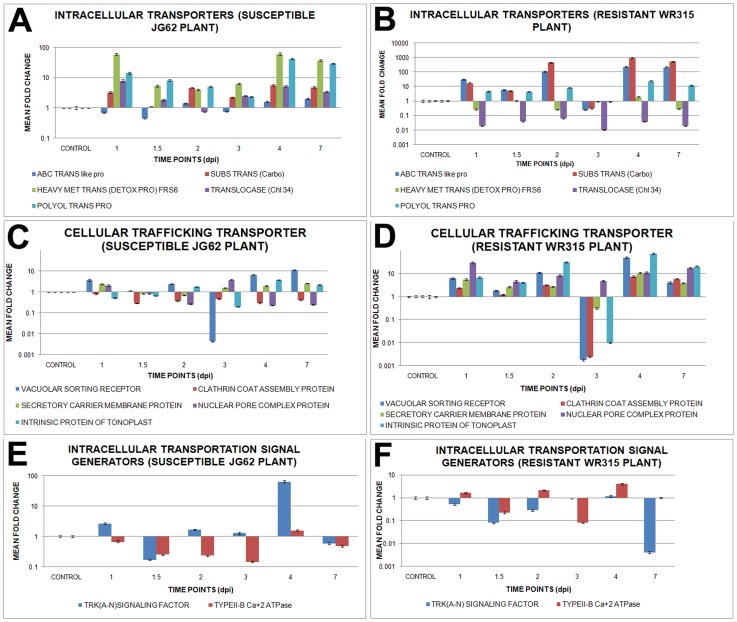
Relative expression of genes related to intracellular transportation in Foc1 infected chickpea roots. (A) and (B) represents relative expression profile of intracellular transporters like, ABC transporter like protein, carbohydrate substrate transporter, heavy metal transporter (detoxifying protein) (FRS6), translocase (chloroplast 34) and polyol transporter protein in uninduced and induced JG62 and WR315 roots respectively; (C) and (D) represents relative expression array of cellular trafficking transporters like, vascular sorting receptor, clathrin coat assembly protein, secretory carrier membrane protein, nuclear pore complex protein and intrinsic protein of tonoplast in uninduced and induced JG62 and WR315 roots respectively; (E) and (F) shows relative expression pattern of intracellular transportation signal generators like, TRK (A–N) signaling factor and type II-b calcium ATPase in uninduced and induced JG62 and WR315 roots respectively. Bars represent the standard errors (n = 3).

Fungal penetration led to the activation of several signal transporters whose expression profiles were analyzed with pathogen migration. Both intracellular transporters, such as ABC transporter like protein and carbohydrate substrate transporters, showed enhanced expression throughout the study period in WR315 plants, with only plants of 3dpi showing a decrease in expression. However, metal transporter (FRS6), translocase (chloroplast 34), and polyol transporter showed reverse profiles at all time points in resistant WR315 plants compared to susceptible JG62 plants (Fig. 8A and B, Fig. S4).

Besides, expression analyses performed with several cellular transporters, such as vacuolar sorting receptor, clathrin coat assembly protein, secretory carrier membrane protein, nuclear pore complex protein, and intrinsic protein of tonoplast, showed upregulation in resistant WR315 plants compared to susceptible plants during nearly all infective stages. However, plants at 3dpi again showed an opposite expression profile (Fig. 8C and D, Fig. S4).

TRK(A–N) signaling factor showed enhanced expression in susceptible JG62 plants compared to resistant WR315 plants, while type IIB calcium^2+^ ATPase showed mild expressional undulations surrounding basal expression values in both plants, which peaked at 4dpi in resistant plants (Fig. 8E and F, Fig. S4).

Amongst the selected cellular transporters four, such as polyol transporter 5 (PLT5) (homologue of polyol transporter protein), vacuolar sorting receptor 1 (VSR1) (homologue of vacuolar sorting receptor), calcium ATPase 2 (ACA2) (homologue of Type II B calcium^2+^ ATPase), and intrinsic protein of tonoplast 2 (TIP2) (homologue of intrinsic protein of tonoplast) were located in the signaling pathway. PLT5 and ACA2 were found to be linked to autoinhibitory H+ ATPase (AHA10), while VSR1 and TIP2 served as independent nodes. PLT5 and ACA2 were linked to sugar and amino acid transport, respectively. VSR1, associated with vacuolar transport, was found to regulate plant stress. ACA2 was also found to negatively influence the expression of calcium-dependent protein kinase (CDPK), while TIP2 was known to downregulate MAP kinase (Fig. S6).

### Foc1 activates expression of transcription factors

**Figure 9:**
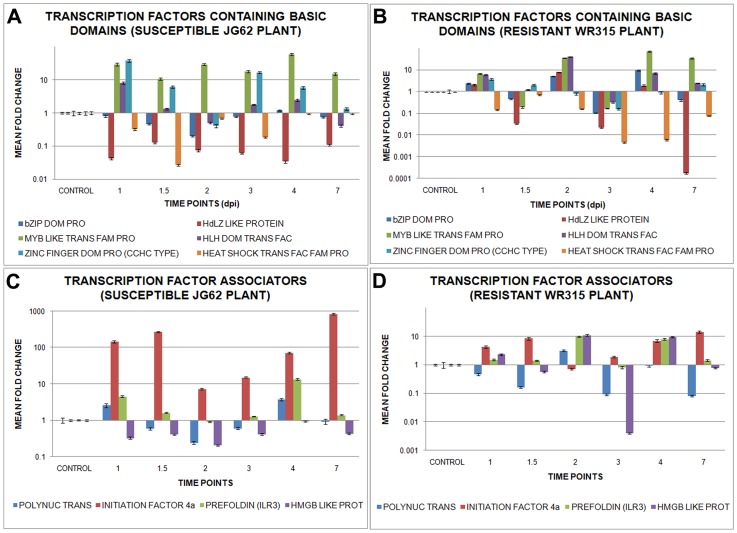
Relative expression of Foc1 induced transcription factors in chickpea roots. (A) and (B) represents relative expression profile of transcription factors containing basic domains like, bZIP domain containing transcription factor, homoeodomain leucine zipper transcription factor, MYB domain containing transcription factor, helix loop helix motif bearing transcription factor, zinc finger (CCHC type) transcription factor and heat shock transcription factor family protein in uninduced and induced JG62 and WR315 roots respectively; (C) and (D) shows comparative expression pattern of transcription factor associators like, polynucleotidyl transferase (FAR1), initiation factor (4a), prefolding helix loop helix domain containing binding factor (ILR3) and high mobility group B like protein in uninduced and induced JG62 and WR315 roots respectively. Bars represent the standard errors(n = 3).

Several TFs with domains such as bZIP, homeodomain leucine zipper, MYB, helix loop helix, zinc finger (CCHC type), and heat shock family protein showed differential expression post Foc1 infection. Expression of both bZIP domain containing TF and homeodomain leucine zipper like protein showed expressional changes in both plants during all time points of the assay. However, MYB domain containing protein showed enhanced expression in susceptible JG62 plants at all time points except for 2dpi, 4dpi and 7dpi where expression of resistant WR315 plants superseded that of susceptible JG62 ones. In case of helix-loop-helix motif bearing TF, enhanced expression was observed in susceptible JG62 plants immediately at 1dpi, which fell close to basal levels at later time points; whereas in resistant plants, alternate rise and drop in expression were evident. Zinc finger protein (CCHC type) showed significant changes at nearly all time points except for 2dpi and 7dpi in susceptible JG62 plants, whereas heat shock factor family protein showed nearly basal expression values for both plants (Fig. 9A and B, Fig. S4).

Transcription factor associated proteins, such as polynucleotidyl transferase (FAR1) showed comparably higher expression in susceptible JG62 plants compared to resistant plants, except at 1.5dpi and 2dpi, for which low expression was observed. Both initiation factor (4a) and the helix-loop-helix motif containing prefoldin (ILR3) showed enhanced expression in susceptible JG62 plants, while the high mobility group B like protein showed upregulation in resistant WR315 plants alternately at 1dpi, 2dpi, and 4dpi (Fig. 9C and D, Fig. S4).

Interaction network showed the location of five genes, such as zinc finger protein (CCHC type) 2 (AZF2) (homologue of zinc finger protein CCHC type), homeodomain leucine zipper REV (homologue of homeodomain leucine zipper like protein), heat shock factor 3 (HSF3) (homologue of heat shock factor family protein), high mobility group protein B3 (HMGB3) (homologue of high mobility group B like protein), and MYB (homologue of MYB domain containing protein) in the defense regulatory pathway. All TFs showed independent nodal positions. MYB negatively regulated apoptosis, while REV positively regulated lignification, auxin mediated polar transport, growth, and cell fate. REV was also found to regulate microRNAs, proteasome degradation, and other homeobox leucine zippers. AZF2 and HMGB3 showed stress mediated regulation. HSF3 downregulated peroxidases (Fig. S7).

### Role of sugar metabolism in defense

**Figure 10:**
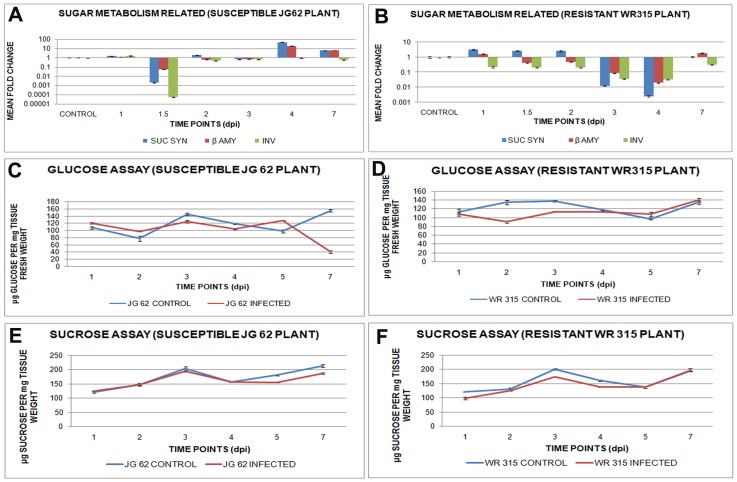
Expression and content of sugar metabolizing genes and sugars in Foc1 infected chickpea roots. (A) and (B) represents relative expression pattern of sugar metabolizes like, sucrose synthase, beta amylase and invertase in uninduced and induced JG62 and WR315 roots; (C) shows relative content of glucose in uninduced and induced JG62 plants; (D) represents relative content of glucose in uninduced and induced WR315 plants; (E) represents relative amount of sucrose in uninduced and induced JG62 plants; (F) shows relative amount of sucrose in uninduced and induced WR315 plants. Bars represent the standard errors (n = 3).

Sugars are known to play crucial roles in defense and are metabolized by several enzymes, such as sucrose synthase, β-amylase, and invertase. Expression of these sugar metabolizers were found to undulate with pathogen progression. Expression of sucrose synthase increased at early time points in resistant WR315 plants while their levels increased significantly after 4dpi in susceptible JG62 plants. Both β-amylase and invertase showed increased expression in susceptible JG62 plants compared to resistant ones throughout pathogen invasion and establishment (Fig. 10A and B, Fig. S4).

Sucrose and glucose, which are known as signal generators, showed post infection metabolic undulations. Glucose and/or sucrose contents reportedly vary during different developmental stages of plant life. Hence, relative amounts of these sugars were monitored along with pathogen progression. Glucose levels showed alterations with an increase at 5dpi, which was met with an abrupt reduction at 7dpi in JG62 plants (Fig. 10C) while WR315 plants showed the same profiles for both glucose and sucrose (Fig. 10D). Sucrose content was comparable with that of uninduced controls up to 4dpi, but was found to reduce at 5dpi and 7dpi in JG62 plants (Fig. 10E). On the contrary, in WR315 plants, the pathogenic response though was obvious at as early as 1dpi but sucrose levels reached to comparable amounts with that of uninduced controls at 5dpi onwards (Fig. 10F).

### Foc1 induced redox regulators, cellular transporters, and transcription factors interconnect in the interaction network

Network analyses performed using redox regulators, cellular transporters, and transcription factors as inputs resulted in an integrated signaling network showing interactions between the three classes of components described above. Redox regulators, namely RBOHF and OCP3, were found to interact with the cellular transporter TIP2 and the TF AZF2, with the small molecule abscisic acid (ABA) acting as a common mediator. The TF AZF2 showed links with FSD1 through other zinc finger protein (STZ). VSR1, found as regulator of vacuolar transport, was linked to auxin responsive transcription factor (ARF). JA appeared to be a common modulator of redox responsive OCP3 and cellular transporter TIP2. RBOHF was found to be regulated by ethylene, ROS, and H_2_O_2_, with the latter acting as a common regulator of TRX. Iron regulated FRO7, while different sugars and H^+^ appeared to regulate PLT5, ACA2, and AHA10. Galactose sugars as well as by lipids regulated VSR1. Apart from ABA and JA, OCP3 was also found to be positively influenced by salicylic acid and methyl jasmonates. ATP, copper, and calcium regulated ACA2, while flavin mononucleotide regulated FQR1. RBOH, a positive regulator of hypersensitive response and cell death, was also found to act as an indirect regulator of cell cycle through TRX3 connections and of defense response through OCP3 links. RBOH was also found to interact with calcium dependent serine threonine kinase (OST1) and other RBOH isoforms (RBOHD). TRX3 showed a negative regulatory relationship with glutaredoxins (GRX4and MPO 12.80) and thioredoxin reductase B (NTRB). Additionally, FRO7 was interlinked with the outer envelope membrane protein OEP7 and the photosystem II reaction center. Cytochrome b5 reductase was found to negatively regulate cytochrome b5. VSR1 regulated syntaxin signaling, while ACA2 influenced the sodium transporter. AZF2 showed interactions with lipoxygenases and the jasmonate domain containing protein (JAZ1). Among other TFs, REV showed interactions with other leucine zippers, while HSF3 showed negative regulatory connections with peroxidase (APX1) and was positively linked with galactinol synthase (GOLS1) (Fig. S8).

### Sugar metabolizers interact with the fungal induced redox regulators, cellular transporters, and transcription factors in the interaction pathway

Sucrose synthase (SUS4), β-amylase (BAM1), serine threonine kinase (CDKB1.1), and vacuolar ATPase (TUF) were reported to play role in modulating sugar metabolism upon Foc1 invasion [27], [32]. These molecular candidates also showed interactions with differentially expressed redox responsive candidates, cellular transporters, and transcription factors analyzed in the present study. RBOH was found to be directly linked with CDCKB1.1 and OCP3 through ABA and indirectly linked with TUF and BAM through CDCKB1.1. A relationship between TRX3 and the above molecular candidates was also observed through RBOH and H_2_O_2_ small molecules. SUS4 and BAM1 appeared as direct regulators of sugar metabolism, while TUF and CDCKB1.1 directly regulated cell differentiation and plant development. Additionally, SUS4 regulated cell wall biosynthesis, turgor pressure, and glycolysis, while CDCKB1.1 regulated cell size and mitotic entry (Fig. S9). Interaction pathways involving sugar-metabolizing genes and cellular transporters showed direct communication between SUS4, PLT5, and ACA2, with d-glucose and ATP acting as intermediate small molecules. CDCKB1.1 showed links with TIP2 through the modulator ABA (Fig. S10). CDCKB1.1 showed direct communication with AZF2, with ABA acting as a mediator. Embryonic development appeared to be the common interacting process for both TUF and REV, while plant development appeared to be common for BAM1 and AZF2. REV also showed a direct interaction with CDCKB1.1, with cytokinesis as an intermediate connecting cell process (Fig. S11).

## Discussion

Previous studies conducted on chickpea–Foc1 interaction have already documented novel insights, which are considered deviations from classical concepts, where HR coupled to PCD promoted fungal colonization and establishment at the xylem vessels of compatible hosts. On the other hand, resistant plants known for early pathogen perception triggered cellular reprogramming, comprising a series of downstream defense responses, all aiding to the diversion of the oxidative burst from the main solute conducting strand, the xylem vessels [27], [32]. Besides, reports of several non-canonical genes lacking a defensive history were also reported to be associated with the present pathosystem [29]. However, with all the previous reports taken together, Foc1 is known to deploy a stealthy “modus operandi” when entering compatible hosts through the breaches of root and root hairs and colonizing xylem vessels. Such diplomatic entry results in an oxidative burst, leading to vessel clogging, blocking upward translocation of essential solutes, all of which manifests into the common symptom of wilt [27]. *Fusarium oxysporum*, though primarily known for its resemblance with biotrophs, has recently been argued to hold necrotrophic features also [43]. However, a lack of information regarding functional effector toxins promoting *in planta* necrosis from Foc prevents conclusive classification. Moreover, studies reviewed by Oliver and Ipcho [44] suggest gradual erosion of such nomenclatural dogmas related to classification properties, with *Phytopthora infestans* placed in all three classes of biotrophs, necrotrophs, and the more specialized hemibiotrophs.

Successful penetration is regulated by the ability of the pathogen to breach the initial barriers of cell wall appositions and cell membrane [45]. Recent report showed the progression and differential colonization pattern of *Fusarium oxysporum* f. sp *ciceris* race 0 and race 5 both in compatible and in incompatible hosts [46]. However, it should be noted that, significant variability between the pathogenic races of *Fusarium oxysporum* f. sp *ciceris* show marked difference in their infection and colonization patterns which largely depends on inoculum densities as well as edaphic factors [47, 48.]. In the present study, Foc1 was found to gradually progress and colonize the compatible host, which was marked by the presence of extensive fungal ramification and deposition of callose-degraded products at the xylem vessels. Besides, establishment of a pathogen is marked by its ability to reproduce within the host interior, thus providing a constant flush of pathogenic compounds [49]. In the present study, presence of microconidia within the xylem vessels of susceptible hosts indicated stable establishment of Foc1. Callose, a homopolymer of linear β-1,3-glucose residues with some β-1,6 branches, is known to aid penetration resistance by forming a special permeability barrier during fungal ingress [50]. In contrast, these short-lived callose molecules are degraded by β-1, 3-glucanases, which are also known to induce pathogen invasion [51]. Previous studies have revealed induction of β-1,3-glucanases in chickpea during compatible interaction with Foc1, suggesting that callose degradation is associated with fungal penetration [28]. Present results also show callose-degraded products at the xylem vessels of susceptible JG62 plants along with high levels of β-1,3-endo glucanase at later stages of infection, corroborating the results of earlier reports. However, the observation of resistant WR315 plants with overall low β-1,3-glucanase levels showing no such degradations even after 7dpi, when wilting symptoms are prominently set in susceptible plants, indicates the use of a different reprogramming mechanism that prevents functional callose catabolism during incompatible interactions. However, an increase in β-1,3-endo glucanase expression in resistant plants at 3 dpi indicates a transient pathogenic attempt to overpower the host defense.

Pathogen invasion leads to oxidative burst and generation of active oxygen species at the site of entry, causing lipid peroxidation and membrane damage, which are believed to be key features of pathogenesis [52]. In the present study, lipid peroxidation mediated membrane damage was evident with gradual pathogen progression in susceptible plants; resistant plants showed a marginally low degree of lipid peroxidations during later stages of infection. These results were found to validate earlier reports [36]. Interestingly, in the present study, assays indicating membrane damage showed cell shrinkage and gradual nuclear adpression in response to pathogen progression in both compatible as well as incompatible host cells, with the degree of nuclear adpression being marginally lower in the resistant host. Previous studies involving *Vigna unguiculata* and *Uromyces vignae* showed similar trends of nuclear migration followed by the cessation of cytoplasmic streaming and alterations in plasma membrane permeability [53]. Changes in nuclear structure and organization were also reported in *Medicago truncatula* and *Daucus carota* during colonization by arbuscular mycorrhizal fungi [54]. However, in the present case study, in compatible interactions such nuclear migration was assumed to be the morphological feature indicating gradual PCD [55], but in case of incompatible interactions in which PCD was not evident, why such nuclear migration occurred was unclear. However, it opens up an exciting arena of future research where nuclear structural and organizational alterations could probably be assigned as signs of cellular reprogramming instead of being termed as signatories of PCD.

Transcript profiling is known to provide details regarding pathogen induced gene expression in hosts, particularly when complete genome datasets and their annotations are limited [56]. In the present study, attempt was made to provide logical interpretation of the defense signaling network occurring within chickpea using transcript profiling. Pathogen invasion is sensed by host cells by triggering an initial alteration in its redox state of art [57].

**Figure 11:**
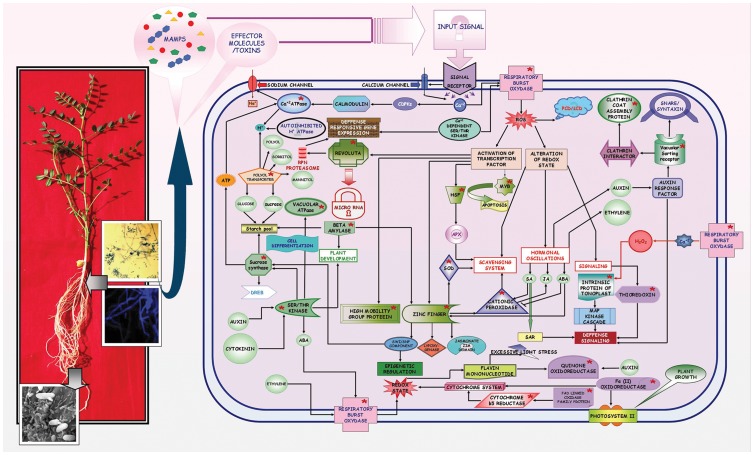
Schematic representation of Foc1 induced defense signalling network in chickpea. Interaction map showing intracellular signalling involving defense responsive molecular components (redox regulators, cellular transporters, transcription factors and sugar metabolizers; marked in red colored asterisks) in chickpea upon Foc1 induction.

Previous studies performed on chickpea by our group as well as by other groups reported the involvement of several transcripts regulating the redox state during onset of Foc1 infection [27], [29], [32], [36]. But, how their expression profiles altered with the gradual pathogen progression was elusive. The present study was focused at understanding the differential expression of selectively those transcripts that were reported to have associations with chickpea-Foc1 casestudy (Fig. 11).

Network analysis showed the location of RBOHF, OCP3, FSD1, TRX3, FRQ1, CBR and FRO7 in the interaction network. RBOHs are known to generate reactive oxygen species (ROS), promote PCD, and trigger downstream hormonal fluctuations upon pathogenic attack [58]–[59]. RBOH isoforms are also known to induce penetration resistance to *Blumeria graminis* f. sp. *hordei* in barley and mediate SAR in *Arabidopsis thaliana* [60]–[61]. In the present study, alteration of RBOH in resistant plants suggests that it has a regulatory role during Foc1 invasion, while susceptible plants failed to show similar variations. OCP and FSD are also known to play key roles in ROS signaling. OCP, an ABA-dependent transcriptional regulator, was suppressed during necrotrophic pathogen attack in *Arabidopsis thaliana* [62], while cationic peroxidase itself and found to accumulate in xylem vessels of *Xanthomonas oryzae* p. v. *oryzae* infested rice plants [63]. FSD is known to serve as the first line of defense against ROS injury [64]. In the present study the sequential undulations of OCP and FeSOD/FSD suggests the scavenging machinery to be under constant functioning during Foc1 infection. RBOH was also found to regulate expression of TRX, which in turn regulate the redox state of other target proteins and participate in oxidative stress tolerance of plants [65]. The interaction of Cf9 protein with TRX was found to negatively regulate defense in tomatoes upon *Cladosporium fulvum* attack [66]. Though results obtained in the present study also showed an overall enhanced expression of F type thioredoxin in compatible interaction compared to incompatible one, but whether it plays a similar negative regulatory role in the present case study needs to be further introspected. FQR1 acts as detoxifier along with GST [67]. CBR is known to catalyze the transfer of electrons from NADH to membrane bound cytochrome b5 reductase [68]. Besides, they also participate in the desaturation reaction during fatty acid synthesis [69]. Fatty acids are known to play important roles in basal, effector-triggered and systemic immune responses [70]. Disappearance of CBR at 3dpi in resistant plants suggests a transitory attempt of the fungus to overpower the resistant host machinery, which probably failed at later hours of infection. On the other hand, FRO7 known to play essential role in delivering iron to chloroplasts [71] showed an average increase in expression at 1dpi and 4dpi in susceptible plants. Such increment probably indicated an attempt of the susceptible host to shield its photosynthetic apparatus that was found to undergo rapid chlorosis upon pathogen progression (Fig. S5, Fig 11).

Apart from the above, in the present study transcriptomic analysis showed enhanced expression of ROS scavenger, peroxidase in resistant plants probably indicating timely elimination of ROS [72]. GSTs, known to reduce organic hydroperoxides formed during oxidative stress were induced in *Nicotiana benthamiana* following *Colletotrichum destructivum* infection [73]. Hence, low expression of GST (TAU26) in WR315 plants probably indicated an overall low concentration of oxidative stress intermediates in resistant plants. In contrast, FAD oxidase family protein known to use molecular oxygen to form disulphide bonds associated with generation of hydrogen peroxides [74], showed enhanced expression at later time points in susceptible plants. Increment of FAD oxidase family protein in susceptible plants probably indicated pathogen triggered generation of peroxides. NADP oxidoreductase helps in regulating the electron partitioning in chloroplast [75]. Sharp decline of NADP oxidoreductase in resistant plants at 3dpi was assumed to be a transient pathogenic attempt to overpower resistant host machinery. H^+^ ATPases are reported to be under dynamic spacio-temporal regulation during early stages of pathogen recognition. Besides, they are also known to control the exposure and closure of stomatal aperture [76]. In the present study, upregulation of H^+^ ATPases throughout in resistant plants suggested early pathogen perception as well as regulation of stomatal opening which is believed to have a direct negative influence on causing wilt (Fig 11).

The signals of altered redox state are communicated to the cell interior through several cellular transporters (Fig. S6, Fig 11). Network analysis showed the integration of PLT5, VSR1, TIP2 and ACA2 in the signaling network. PLT5 plays pivotal role in transporting sugars primarily to non-photosynthetic tissues such as the roots [77]. Researches conducted on *Arabidopsis thaliana* showed that sugars assist the symport of substrates such as myo-inositol, glycerol, and ribose sugar [78]. Results obtained from the present study showing enhanced expression of PLT in susceptible plants suggested the requirement of soluble sugars and its transportation to be probably under pathogenic regulation instead of being governed by the host. Vacuolar sorting receptor (VSR), which is known for proper targeting of soluble cargo proteins to destination compartments, was found to regulate plant stress in *Arabidopsis thaliana* through ABA mediated signaling [79]. In the present study, VSR showed enhanced expression in incompatible reactions, suggesting intracellular transportation during pathogen attack, with only plants at 3dpi showing an unexplainable exceptional profile. TIP, a specialized aquaporin located in the tonoplastic membrane, regulates vacuolar transport and activates MAP kinases linked to defense. Researches conducted on *Arabidopsis* showed downregulation of TIP during water and oxidative stress [80]. Interestingly, in the present study, susceptible plants showed an average low expression of TIP throughout pathogen progression, further validating oxidative injury that resulted in xylem clogging and water stress. ACAs are also thought to regulate salt stress along with calcium dependent protein kinases (CDPKs) and calmodulin, which act as negative regulators [81]–[82]. Present study showed enhanced expression of ACA in resistant plants, suggesting a role for CDPKs and calmodulin in host defense.

Besides, transcriptomic profile indicated ABC transporter like protein to be upregulated in the incompatible interaction in the present study. These transporters showed enhanced expression during fungal invasion in *Arabidopsis thaliana* and in the presence of SA and methyl jasmonate in soybean [83]–[84]. Additionally, PDR-type ABC transporter was known to mediate cellular uptake of the stress-regulating hormone ABA [85]. Carbohydrate substrate transporters are known to have complex roles in plant defense. On one hand, they are believed to fight invasion, while on the other they are also known to be hijacked by the invaders [86]. In the present study, enhanced expression of carbohydrate substrate transporters in incompatible interaction probably indicated its role in preventing fungal invasion. Translocase (chloroplast 34) regulates GTPase mediated protein import of chloroplasts [87]. However, in the present study, upregulation of translocase (chloroplast 34) in susceptible plants does not describe any specific role. Clathrin coat assembly proteins and secretory carrier membrane proteins, though ill characterized in plants, are known to aid exocytosis in association with SNARE proteins and syntaxins [88]–[89]. Enhanced expression of these secretory proteins in resistant plants indicates that exocytosis is related to pathogenesis. Recent studies have highlighted the significance of nucleocytoplasmic transport involving nuclear pore complex proteins in plant defense [90]. Results obtained in the present study showed distinct upregulation of nuclear pore complex proteins in resistant plants, suggesting that nucleocytoplasmic transport is coupled with defense responses against Foc1 (Fig 11).

Under stressful situations transcription factors are believed to act as prime expressional regulators of defensive genes [91]. Signaling network showed the presence of AZF2, REV, MYB, HSF3 and HMGB transcription factors. Zinc finger (CCHC type) transcription factor, which are known for their roles as repressors during drought, cold, salt, and oxidative stresses, are known to regulate jasmonate signaling, expression of lipoxygenase, and switch/sucrose non-fermentable (SWI/SNF) components, which are further known to regulate epigenetic responses [92]–[94]. Results of present study showing decreased expression of zinc finger protein (CCHC type) in incompatible host validated the repressive role of zinc finger protein (CCHC type) under fungal induction. REV, a homeodomain leucine zipper like protein, showed expressional changes in the present study. Similar variation was observed in sunflowers during wounding and biotic stress [95]. MYB domain containing TF, a regulator of HR, biotic stress and salt tolerance in *Arabidopsis thaliana* [96]–[98], was found to show fluctuations at early time points of Foc1 infection which reached stable expressional levels at later time points in resistant plants. The reason behind such fluctuations needs to be investigated. However, the susceptible plants showed a steady level of expression probably indicating cellular apoptosis by Foc1 infection. HSFs function as ROS sensors by sensing hydrogen peroxides in particular [99]. Such heat shock factor family proteins were found to be downregulated in resistant plants, suggesting low levels of ROS. HMG are reported to be upregulated under salinity stress, drought or cold stresses [100]. The present study showing enhanced amounts of HMG B like protein in resistant plants probably indicated similar stress regulatory function (Fig. S7, Fig 11).

Additionally, helix-loop-helix and bZIP domain bearing transcription factors, known for mediating jasmonate specific responses in *Arabidopsis thaliana* [101] also showed differential expressional undulations in the present study (Fig. 11). The role of poly nucleotidyl transferase that is known to have exonuclease activity needed for DNA repair [102] is unclear in the present study. Translational initiation factor 4a is reported to be the prototype of DEAD box family protein that helps in initiation of translation [103]. However, understanding the role of this factor in the present study requires additional experimentation. Prefoldin is reported to be essential for microtubule assembly [104]. Increased levels of prefoldin in susceptible plants probably indicated microtubule assembly which is thought to be an essential mechanism for cell repair.

Sugars act as signal transducing molecules that are known to integrate defense signal transduction. Previous reports have documented the role of sucrose synthase, β-amylase, vacuolar H^+^ ATPase, and serine threonine kinases in regulating defense signaling [27], [32]. Network analysis showed interactions between these previously described sugar modulators and the redox responsive transcripts, cellular transporters and transcription factors discussed in the present study. These soluble sugars are known to actively participate in oxidative stress regulation [105]. Additionally, sucrose synthase levels are found to elevate during cold acclimation in *Arabidopsis thaliana*, with DREB acting as the transcriptional activator behind this elevation [106]. Sucrose synthase is also known to regulate several serine threonine kinases, which are in turn found to be under hormonal control [107]. These serine threonine kinases, along with β-amylase, directly control plant development, while both H^+^ ATPase and serine threonine kinase control cell differentiation, golgi organization, and vacuolar function [108]–[109]. Although, in the present study, during compatible interaction, the levels of soluble sugar metabolizing genes such as sucrose synthase, β-amylase, and invertase were found to be sharply downregulated at 1.5dpi, but showed gradual expressional increment after 2dpi, with sucrose synthase and β-amylase showing expressional peaks at 4dpi. However, a decrease in the net amount of sucrose as well as its conversion product glucose in susceptible plants observed at later time points of infection, suggested expenditure of the released hexoses to be more pathogen driven than host regulated. This result supports the previous report on *Ustilago maydis*, where the host-derived hexose sugars were utilized to fuel the primary metabolism of the invading fungus [110]. *Pseudomonas* spp. and *Xanthomonas* spp. were also known to induce SWEET, a glucose uniporter from *Arabidopsis thaliana*, to drive self metabolism [111]. In the present case study resistant plants probably reorient their primary metabolism to satisfy optimum self demands while regulating the resources needed to prevent and repair fungal induced cell injury (Fig. S9, S10, S11, and Fig. 11).

## Conclusion

Considering all the illustrations and explanations, the interaction network generated from the transcriptomic profiling and pathway analyses was an attempt to delineate the probable defense signaling network found in chickpea as an outcome of Foc1 challenge (Fig. 11). The challenge led to the colonization and establishment of the fungus in susceptible host, while the resistant host could judiciously reprogram its metabolism in warding off pathogenic consequences. However, a remarkable decrease in the expression of redox related transcripts such as ROS generators and scavengers as well as cytochrome dependent redox signal transducers were found at 3dpi in the incompatible host. Apart from these, intracellular transporters, basic domain containing TFs, and sugar metabolizing genes were also downregulated. These declines in expression levels suggest a pathogenic effort to overwhelm the resistant host defense, which collapsed at later time points. RBOH, OCP, VSR, PLT, SUS, serine/threonine kinase, and zinc finger protein (CCHC type) appeared to be key molecular candidates controlling important hubs of the defensive network. Functional characterization of these hub controllers may provide promising clues for further understanding of the chickpea–Foc1 interaction. Additionally, it could also help in developing this case study into a model for investigating the intricacies of vital vascular diseases such as wilt, in which the host must defend against pathogen establishment while maintaining normal metabolic homeostasis. Recent availability of the draft genome sequence of chickpea (*Cicer arietinum* L.) is not only expected to add to the knowledge of chickpea-Foc1 interaction, but also aid to the development of effective disease management strategies [112].

## Supporting Information

Figure S1 **Confocal scanning laser microscopic images representing pathogen induced tissue damage.** (a–d) corresponds to root sections of uninduced JG62 plants; (e–h) corresponds to root sections of uninduced WR315 plants. (i–l) represent root sections of infected JG62 plants at 12 dpi; (m–p) represent root sections of infected WR315 plants at 12dpi. (a,e,i,m) show fluorescent images stained with sytox green; (b,f,j,n) show fluorescent images stained with propidium iodide; (c,g,k,o) represent differential interference contrast (DIC) images; (d,h,l,p) represent merged images. Bars represent 20 µm.
(PDF)

Figure S2 **Graphical representation of biochemical assayof pathogen induced lipid peroxidation in JG62 and WR315 plants.**
(PDF)

Figure S3 **Confocal Scanning Laser Microscopy images showing measurement of nuclear adpression during pathogen progression.** (a) Uninduced JG62 root cell. (b and c) Induced JG62 root cells at 7dpi and 12dpi respectively. (d) Induced WR315 root cell at 12dpi. Bars represent 20 µm.
(PDF)

Figure S4 **Heat map showing differential levels of redox regulators, cellular transporters and transcription factors induced in JG62 and WR315 plants after Foc 1 infection.**
(PDF)

Figure S5 **Network showing pathogen induced intracellular redox signaling.**
(PDF)

Figure S6 **Network showing pathogen induced intracellular signal transportation.**
(PDF)

Figure S7 **Network showing transcription factors and associated signaling.**
(PDF)

Figure S8 **Network showing interaction between redox regulators, cellular transporters and transcription factors.** ATCBR, *Arabidopsis thaliana* NADH cytochrome b5 reductase; HSF3, heat shock factor 3; FQR1, flavodoxin like quinone reductase1; FSD1, iron superoxide dismutase; STZ, cys2/his2 zinc finger; AZF2, zinc finger (CCHC type); VSR1, vacuolar sorting receptor1; ATRBOH, *Arabidopsis thaliana* respiratory burst oxidase homologue; FRO7, ferric reduction oxidase 7; OCP3, over expression of cationic peroxidase 3; HMG3, high mobility group B protein 3; PLT5, polyol transporter protein 5; AHA10, autoinhibited H^+^ ATPase isoform 10; ACA2, calcium ATPase; MYB106, MYB transcription factor 106; REV, homoeobox leucine zipper (REVOLUTA); TRX3, thioredoxin 3; TIP2, tonoplast intrinsic protein 2.
(PDF)

Figure S9 **Network showing interaction between sugar metabolizers and redox regulators. SUS4, sucrose synthase 4; BAM1, beta amylase; CDKB1.1, serine threonine kinase; TUF, vacuolar ATPase.**
(PDF)

Figure S10 **Network showing interaction between sugar metabolizers and cellular transporters.**
(PDF)

Figure S11 **Network showing interaction between sugar metabolizers and transcription factors.**
(PDF)

Table S1 **Excitation and emission wave lengths of fluorescent dyes.**
(DOC)

Table S2 **Values of error of biochemical assay indicating lipid peroxidation.**
(XLS)

Table S3 **Database homology matches of chickpea genes used in the study.**
(DOC)

Table S4 **Primer sequences with database references used for qRT PCR.**
(DOC)

Table S5 **Mean fold change, standard deviation and standard error of the transcripts used for qRT-PCR analyses.**
(XLS)

Table S6 **Statistical t and p value calculated by Student's t test of the transcripts used for qRT-PCR analyses.**
(XLS)

Table S7 **Values error calculated during sugar assay.**
(XLS)
