# Supplementary figures and images for: Fusarium oxysporum f.sp. ciceri Race 1 Induced Redox State Alterations Are Coupled to Downstream Defense Signaling in Root Tissues of Chickpea (Cicer arietinum L.)
Source: PLoS One. 2013 Sep 13;8(9):e73163. doi: 10.1371/journal.pone.0073163 (PMC3772884; doi:10.1371/journal.pone.0073163)

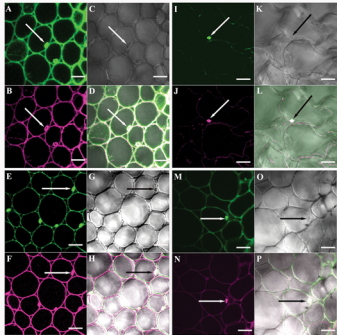

Supplement: Figure S1 — Confocal scanning laser microscopic images representing pathogen induced tissue damage. (a–d) corresponds to root sections of uninduced JG62 plants; (e–h) corresponds to root sections of uninduced WR315 plants. (i–l) represent root sections of infected JG62 plants at 12 dpi; (m–p) represent root sections of infected WR315 plants at 12dpi. (a,e,i,m) show fluorescent images stained with sytox green; (b,f,j,n) show fluorescent images stained with propidium iodide; (c,g,k,o) represent differential interference contrast (DIC) images; (d,h,l,p) represent merged images. Bars represent 20 µm. (PDF) [file pone.0073163.s001.pdf]

## LIPID PEROXIDATION ASSAY

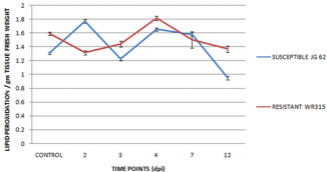

Supplement: Figure S2 — Graphical representation of biochemical assayof pathogen induced lipid peroxidation in JG62 and WR315 plants. (PDF) [file pone.0073163.s002.pdf]

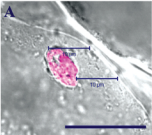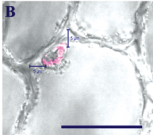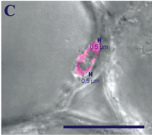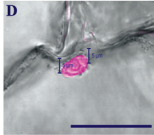

Supplement: Figure S3 — Confocal Scanning Laser Microscopy images showing measurement of nuclear adpression during pathogen progression. (a) Uninduced JG62 root cell. (b and c) Induced JG62 root cells at 7dpi and 12dpi respectively. (d) Induced WR315 root cell at 12dpi. Bars represent 20 µm. (PDF) [file pone.0073163.s003.pdf]

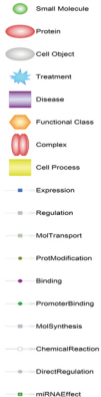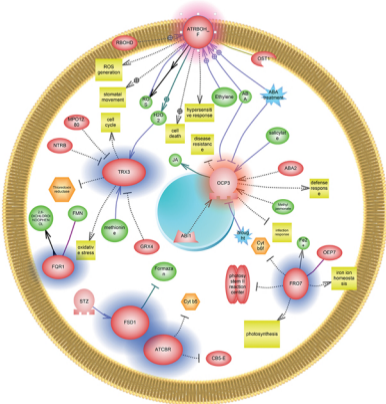

Supplement: Figure S5 — Network showing pathogen induced intracellular redox signaling. (PDF) [file pone.0073163.s005.pdf]

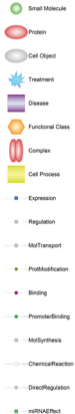

Supplement: Figure S6 — Network showing pathogen induced intracellular signal transportation. (PDF) [file pone.0073163.s006.pdf]

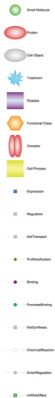

Supplement: Figure S7 — Network showing transcription factors and associated signaling. (PDF) [file pone.0073163.s007.pdf]

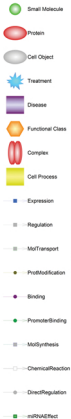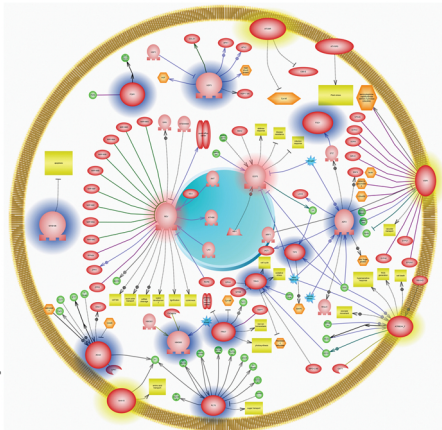

Supplement: Figure S8 — Network showing interaction between redox regulators, cellular transporters and transcription factors. ATCBR, Arabidopsis thaliana NADH cytochrome b5 reductase; HSF3, heat shock factor 3; FQR1, flavodoxin like quinone reductase1; FSD1, iron superoxide dismutase; STZ, cys2/his2 zinc finger; AZF2, zinc finger (CCHC type); VSR1, vacuolar sorting receptor1; ATRBOH, Arabidopsis thaliana respiratory burst oxidase homologue; FRO7, ferric reduction oxidase 7; OCP3, over expression of cationic peroxidase 3; HMG3, high mobility group B protein 3; PLT5, polyol transporter protein 5; AHA10, autoinhibited H+ ATPase isoform 10; ACA2, calcium ATPase; MYB106, MYB transcription factor 106; REV, homoeobox leucine zipper (REVOLUTA); TRX3, thioredoxin 3; TIP2, tonoplast intrinsic protein 2. (PDF) [file pone.0073163.s008.pdf]

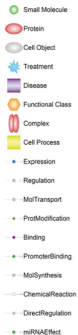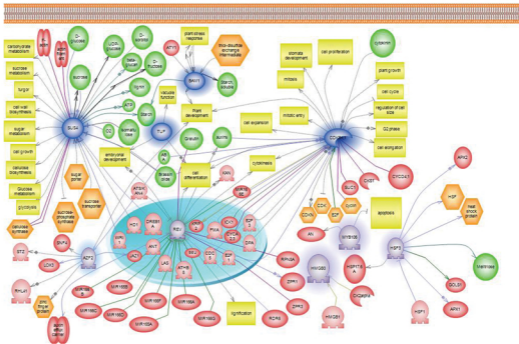

Supplement: Figure S11 — Network showing interaction between sugar metabolizers and transcription factors. (PDF) [file pone.0073163.s011.pdf]
