# Supplementary material for: Fusarium oxysporum f.sp. ciceri Race 1 Induced Redox State Alterations Are Coupled to Downstream Defense Signaling in Root Tissues of Chickpea (Cicer arietinum L.)
Source: PLoS One. 2013 Sep 13;8(9):e73163. doi: 10.1371/journal.pone.0073163 (PMC3772884; doi:10.1371/journal.pone.0073163)
Supplement: Figure S4 — Heat map showing differential levels of redox regulators, cellular transporters and transcription factors induced in JG62 and WR315 plants after Foc 1 infection. (PDF) [file pone.0073163.s004.pdf]

Category	Subgroup	NAME OF THE GENES		Control	1 DPI	1.5 DPI	2 DPI	3 DPI	4 DPI	7 DPI
REDOX SENSITIVE GENES	ROS Generators and scavengers	RESPIRATORY BURST OXIDASE	JG 62	1	2.317201081	1.022868	0.102058	2.606168	2.569439	1.448903
			WR 315	1	8.802505175	0.222817	10.8017	0.400483	57.76088	0.580467
		PEROXIDASE	JG 62	1	5.637363564	9.9134	0.027595	6.509124	87.21136	1.967449
			WR 315	1	31.99419798	16.94811	416.0224	0.391201	216.9203	42.33646
		CATIONIC PEROXIDASE	JG 62	1	19.18668217	1.13102	0.855075	4.879515	0.168611	5.64016
			WR 315	1	0.004101515	0.427646	7.310074	1.032354	15.1125	1.67838
		SUPEROXIDE DISMUTASE	JG 62	1	4.367246035	3.333274	0.057694	1.644602	18.46141	0.658647
			WR 315	1	6.581884872	7.193983	15.63429	0.24894	42.36685	4.760614
		GLUTATHIONE S TRANSFERASE	G 62	1	7.52578159	0.123268	2.96946	0.846313	112.7363	41.92932
			WR 315	1	1.476139987	5.024417	1.330818	0.005682	0.003153	2.103231
	Cytochrome dependent redox signal transducers	CYT b561 Fe REDUCTASE	JG 62	1	1.270100099	0.744794	0.055505	0.604834	37.75228	1.012623
			WR 315	1	7.854155596	5.379024	36.80774	0.784492	113.7568	8.451977
		CYT b5	JG 62	1	0.694702493	0.025758	0.230367	0.33848	16.90311	5.534077
			WR 315	1	3.012316072	2.671729	1.058333	0.082309	0.000659	0.493543
		NADH CYT b5 REDUCTASE	JG 62	1	8.903855913	0.00014	0.604719	0.831588	43.97002	1.327549
			WR 315	1	1.860360662	15.30097	0.212817	0.004413	0.001048	0.058176
	Intracellular ROS signal transducers	NADP OXIDOREDUCTASE	JG 62	1	0.522166501	0.806707	0.304416	2.011074	8.59918	2.154088
			WR 315	1	1.00023597	1.276719	4.118631	0.218403	12.9761	1.050356
		QUINONE OXIDOREDUCTASE	JG 62	1	3.990343808	0.000303	5.580209	1.233734	61.46223	7.523877
			WR 315	1	0.361835606	3.163097	0.272904	0.001727	0.044233	0.063989
		Fe (III) OXIDOREDUCTASE	JG 62	1	25.68233465	3.491527	3.01859	0.67964	55.99826	11.12897
			WR 315	1	0.192649773	0.9125	0.338009	0.733071	0.958959	0.156529
		THIOREDOXIN	JG 62	1	0.005411781	0.000852	1.44957	13.54337	40.0529	31.67976
			WR 315	1	1.61474E-05	0.005734	0.849226	1.613888	0.181345	0.083378
		ATPase	JG 62	1	2.787689276	1.994994	0.064309	8.558084	12.47588	0.580467
			WR 315	1	17.96711478	4.622583	34.12578	0.609468	160.7125	22.43368
GENES INVOLVED IN CELLULAR TRANSPORTATION	Intracellular transporters	ABC TRANSPORTER	JG 62	1	0.70380369	0.456274	1.418366	0.759291	1.623933	2.028307
			WR 315	1	31.61946897	5.909851	111.6382	0.252892	233.7792	215.2738
		SUBSTRATE TRANSPORTER	JG 62	1	3.273845625	1.078277	4.64073	2.241682	5.596056	4.806027
			WR 315	1	17.13934342	5.013525	441.2306	0.313429	954.0221	537.3985
		HEAVY METAL TRANSPORTER	JG 62	1	58.84884887	5.36668	3.977558	6.287706	61.84166	37.76697
			WR 315	1	0.276100227	1.039288	0.25581	0.923254	1.873642	0.291362
		TRANSLOCASE	JG 62	1	7.971129544	1.82911	0.743555	2.476855	5.225048	3.428118
			WR 315	1	0.020252294	0.039743	0.065	0.010408	0.038963	0.020113
		POLYOL TRANSPORTER PROTEIN	JG 62	1	16.27173733	8.134143	5.054486	2.327153	41.68359	29.29154
			WR 315	1	4.593480222	4.436438	8.105408	0.864914	23.71197	11.81583
	Cellular trafficking transporters	VACUOLAR SORTING RECEPTOR	JG 62	1	3.683973278	1.106674	2.432398	0.004352	6.606986	11.38306
			WR 315	1	6.309021711	1.846305	11.11327	0.001779	50.01405	4.088924
		CLATHRIN COAT ASSEMBLY PROTEIN	JG 62	1	0.810574112	0.282464	0.367191	0.453815	0.3014	0.409539
			WR 315	1	2.335454991	1.225724	3.145377	0.002497	7.546343	5.858555
		SECRETORY CARRIER MEMBRANE PROTEIN	JG 62	1	2.373394306	0.836229	0.696994	1.586509	1.957625	2.579699
			WR 315	1	5.654787229	2.637646	2.739328	0.313668	10.69487	3.836251
		NUCLEAR PORE COMPLEX PROTEIN	JG 62	1	2.045541678	0.825693	0.259977	3.830916	0.229408	0.246098
			WR 315	1	29.90416074	4.599182	8.432894	4.77442	11.05808	17.49188
		INTRINSIC PROTEIN OF TONOPLAST	JG 62	1	0.503471478	0.646276	1.78786	0.194581	3.736231	2.177525
			WR 315	1	5.800446829	4.03373	31.16995	0.010047	74.06955	20.78562
	Intracellular transportation signal generators	TRK(A-N) SIGNALING FACTOR	JG 62	1	2.64064531	0.171285	1.688901	1.280588	64.21155	0.594513
			WR 315	1	0.518349811	0.080997	0.283979	0.966848	1.17232	0.004049
		TYPE II-B Ca^{+2} ATPase	JG 62	1	0.672139285	0.253632	0.236381	0.147498	1.58783	0.490322
			WR 315	1	1.655088034	0.227955	2.134697	0.083099	3.999525	1.02176
TRANSCRIPTION FACTORS	Transcription factors containing basic domains	bZIP TRANSCRIPTION FACTOR	JG 62	1	0.807222219	0.460548	0.197842	0.780575	1.202703	0.742099
			WR 315	1	2.337837763	0.453477	5.30597	0.10275	9.226048	0.392229
		HOMEODOMAIN LEUCINE ZIPPER	JG 62	1	0.042731077	0.12702	0.073612	0.061961	0.033807	0.108049
			WR 315	1	2.052112155	0.033418	7.620689	0.02205	1.884619	0.000175
		MYB TRANSCRIPTION FACTOR	JG 62	1	29.14275193	10.8433	29.4542	17.91006	58.39719	15.24618
			WR 315	1	6.41526904	0.190273	34.9334	0.166562	69.37438	34.02996
		HLH TRANSCRIPTION FACTOR	JG 62	1	8.145643954	1.335315	0.506028	1.794565	2.45621	0.409957
			WR 315	1	5.918278773	1.200567	39.35633	0.318465	6.824997	2.427101
		ZINC FINGER	JG 62	1	37.89728087	6.088141	0.414852	16.87636	5.846074	1.324401
			WR 315	1	3.631358432	1.994761	0.785327	0.155916	0.874865	2.096238
		HEAT SHOCK FACTOR	JG 62	1	0.319953193	0.026764	0.681124	0.184182	0.955145	0.983046
			WR 315	1	0.141736216	0.682571	0.156105	0.004539	0.005915	0.075753
	Transcription factor associators	POLYNUCLEOTIDYL TRANSFERASE	JG 62	1	2.596620478	0.589553	0.240797	0.601695	3.712838	0.929605
			WR 315	1	0.458427029	0.161451	3.162938	0.091133	0.934753	0.08256
		INITIATION FACTOR	JG 62	1	144.7184755	267.8444	7.228432	15.03361	71.87749	842.4387
			WR 315	1	4.219970943	8.536797	0.723112	1.914091	6.981904	14.04393
		PREFOLDING HLH BINDING FACTOR	JG 62	1	4.495628253	1.598856	0.898029	1.281501	13.53387	1.383334
			WR 315	1	1.508267189	1.407101	9.924057	0.7998	7.961257	1.44667
		HIGH MOBILITY GROUP PROTEIN	JG 62	1	0.324603668	0.405158	0.20605	0.413395	0.940046	0.432365
			WR 315	1	2.319669451	0.569441	11.03889	0.004014	9.379951	0.77165
SUGAR METABOLISERS		SUCROSE SYNTHASE	JG 62	1	1.5175328	0.001978	1.923031	0.657698	46.81886	5.906638
			WR 315	1	3.172017907	2.686322	2.55516	0.012392	0.002567	1.043224
		β AMYLASE	JG 62	1	1.176189423	0.055426	0.628083	0.708626	18.50489	6.271828
			WR 315	1	1.623722861	0.43556	0.487282	0.088609	0.02037	1.86001
		INVERTASE	JG 62	1	1.606759411	5.43E-05	0.464099	0.670368	0.964292	0.589781
			WR 315	1	0.217511382	0.205124	0.212045	0.03595	0.033075	0.326863

0.5 fold and below — 0.5 to 1 fold — Basal expression (1 to 2 fold) — 2 to 5 fold — 5 fold and above

Basal expression — 0.5 fold and below — 0.5 to 1 fold — 1 to 2 fold — 2 to 5 fold — 5 fold and above
