# Supplementary material for: Fusarium oxysporum f.sp. ciceri Race 1 Induced Redox State Alterations Are Coupled to Downstream Defense Signaling in Root Tissues of Chickpea (Cicer arietinum L.)
Source: PLoS One. 2013 Sep 13;8(9):e73163. doi: 10.1371/journal.pone.0073163 (PMC3772884; doi:10.1371/journal.pone.0073163)
Supplement: Figure S10 — Network showing interaction between sugar metabolizers and cellular transporters. (PDF) [file pone.0073163.s010.pdf]

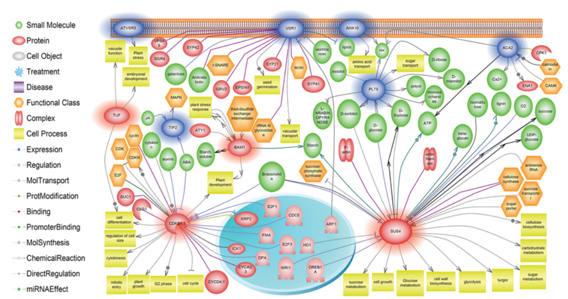

Small Molecule
Protein
Cell Object
Treatment
Disease
Functional Class
Complex
Cell Process
Expression
Regulation
MolTransport
ProtModification
Binding
PromoterBinding
MolSynthesis
ChemicalReaction
DirectRegulation
miRNAEffect
ATVSR3
VSR1
AHA10
ACA2
CPK1
VPS45
SGR4
SYP42
SYP21
SYP41
lectin
t-SNARE
galactose
Anticatabolin
GRV2
EPSIN1
seed germination
vacuole function
Plant stress
embryonal development
TUF
MAPK
plant stress response
thiol-disulfide exchange intermediate
rRNA N glycosidase
vacuolar transport
wortmannin
lipids
amino acid transport
H+
inositol
sugar transport
D-ribose
D-mannitol
PLT5
polyol
monosaccharides
L-ARABINOPYRANOSE
D-sorbitol
D-glucose
D-fructose
ATP
Ca2+
ENA1
calmodulin
CAMK
Isomaltulose
lignin
O2
sucrose
beta-glucan
UDP-glucose
JA
TIP2
ATY1
BAM1
cyclin
CDK
CDKN
cytokinin
auxins
ABA
Starch, soluble
Starch
F-actin
actin filament
sucrose-phosphate synthase
Brassinolide
Plant development
antisense RNA
cellulose synthase
sucrose transporter
sugar porter
E2F
SUC1
CKS1
CDKB1;1
E2F1
CDC5
KRP2
FMA
E2F3
HD1
ICK1
DPA
CYCA2;3
WRI1
DREB1A
ARF1
CYCD4;1
SUS4
cell differentiation
regulation of cell size
cytokinesis
mitotic entry
plant growth
G2 phase
cell cycle
sucrose metabolism
cell growth
Glucose metabolism
cell wall biosynthesis
glycolysis
turgor
sugar metabolism
cellulose biosynthesis
carbohydrate metabolism
