# Supplementary material for: Fusarium oxysporum f.sp. ciceri Race 1 Induced Redox State Alterations Are Coupled to Downstream Defense Signaling in Root Tissues of Chickpea (Cicer arietinum L.)
Source: PLoS One. 2013 Sep 13;8(9):e73163. doi: 10.1371/journal.pone.0073163 (PMC3772884; doi:10.1371/journal.pone.0073163)
Supplement: Table S1 — Excitation and emission wave lengths of fluorescent dyes. (DOC) [file pone.0073163.s012.doc]

| **Supporting Table S1 Excitation and emission wave lengths of fluorescent dyes.** | | |
| --- | --- | --- |
| **NAME OF THE FLUORESCENT DYES** | **EXCITATION MAXIMUM**  **(nm)** | **EMISSION COLLECTION REGION**  **(nm)** |
| **SYTOX Green** | **504** | **510 to 560** |
| **Propidium iodide** | **536** | **610 to 650** |
| **Trypan blue** | **514** | **560** |
| **Aniline blue** | **405** | **505 to 530** |
