# Supplementary material for: Fusarium oxysporum f.sp. ciceri Race 1 Induced Redox State Alterations Are Coupled to Downstream Defense Signaling in Root Tissues of Chickpea (Cicer arietinum L.)
Source: PLoS One. 2013 Sep 13;8(9):e73163. doi: 10.1371/journal.pone.0073163 (PMC3772884; doi:10.1371/journal.pone.0073163)
Supplement: Table S3 — Database homology matches of chickpea genes used in the study. (DOC) [file pone.0073163.s014.doc]

| **Supporting Table S3 Database homology matches of chickpea genes used in the study.** | | | | | | |
| --- | --- | --- | --- | --- | --- | --- |
| **EST NAME** | **EST NUMBERS (NCBI)** | **HOMOLOGIES FOUND** | | **TAIR HOMOLOGUE NAMES** | **PATHWAY CONSTRUCTION** | |
|  |  | **CHICKPEA TRANSCRIPTOME DATABASE (CTDB)** | **OTHER DATABASES** |  | **SOFTWARE GENERATED** | **MANUAL** |
| **1. REDOX RESPONSIVE TRANSCRIPTS** |  |  |  |  |  |  |
| 1. **ROS GENERATORS & SCAVENGERS** 2. Respiratory burst oxidase homologue | CaF1_JIE_03_G_05 | TC16083 | gb|AAW78864.1|  [Medicago truncatula] | ATRBOH_F (AT1G64060) | Pathway generated using literature input from pathway studio (Figure_5_SuppInfo). | Incorporated in manual pathway using input from pathway studio (Figure 10). |
| 1. Peroxidase | CaF1_JIE_03_E_09 | TC04331 | | emb|CAA62226.1| [Medicago sativa] | | --- | | AT2G38380 | No homology found in pathway studio | Incorporated in manual pathway using input from published literature. (Figure 10) |
| 1. Cationic peroxidase | CaF1_JIE_07_G_06 | TC18397 | gb|AAA32676.1|  [Arachis hypogeal] | OCP3 (AT5G11270) | Pathway generated using literature input from pathway studio (Figure_5_SuppInfo). | Incorporated in manual pathway using input from pathway studio (Figure 10). |
| 1. Iron superoxide dismutase | CaF1_WIE_30_B_01 | TC17160 | emb|CAA10160.1|  Cicer arietinum | FSD1 (AT4G25100) | Pathway generated using literature input from pathway studio (Figure_5_SuppInfo). | Incorporated in manual pathway using input from pathway studio (Figure 10). |
| 1. Glutathione S transferase (TAU 26) | CaF1_WIE_50_B_08 | TC01997 | gb|ABG90381.1|  [Caragana korshinskii] | AT1G17190 | No homology found in pathway studio | Incorporated in manual pathway using input from published literature. (Figure 10) |
| 1. **CYTOCHROME DEPENDENT REDOX SIGNAL TRANSDUCERS**   1. Cytochrome b561 Fe reductase | CaF1_WIE_18_D_06 | TC15027 | gb|ABN08458.1|  [Medicago truncatula] | FRO7 (AT5G49740) | Pathway generated using literature input from pathway studio (Figure_5_SuppInfo). | Incorporated in manual pathway using input from pathway studio (Figure 10). |
| 2. FAD linked oxidase family protein | CaF1_WIE_30_F_11 | TC16285 | gb|ABO82384.1|  [Medicago truncatula] | AT4G36400 | No homology found in pathway studio | Incorporated in manual pathway using input from published literature. (Figure 10) |
| 3. NADH cytochrome b5 reductase | CaF1_WIE_24_D_04 | TC14723 | gb|AAV69019.1|  [Vernicia fordii] | ATCBR (AT5G17770) | Pathway generated using literature input from pathway studio (Figure_5_SuppInfo). | Incorporated in manual pathway using input from pathway studio (Figure 10). |
| 1. **INTRACELLULAR ROS SIGNAL TRANSDUCERS** 2. NADP dependent oxidoreductase | CaF1_WIE_33_E_02 | TC15458 | ref|NP_186958.1|  [Arabidopsis thaliana] | AT5G16990 | No homology found in pathway studio | Incorporated in manual pathway using input from published literature. (Figure 10). |
| 1. Quinone oxidoreductase | CaF1_WIE_38_G_09 | TC18358 | gb|AJ487465.1| | FQR1 (AT5G54500) | Pathway generated using literature input from pathway studio (Figure_5_SuppInfo). | Incorporated in manual pathway using input from pathway studio (Figure 10). |
| 1. Fe (II) oxidoreductase | CaF1_JIE_25_A_06 | TC17646 | gb|ABE79228.1|  [Medicago truncatula] | AT5G24530 | No homology found in pathway studio | Incorporated in manual pathway using input from published literature. (Figure 10). |
| 1. F-type thioredoxin | CaF1_WIE_04_C_09 | TC18037 | sp|P29450|TRXF_PEA  [Pisum sativum] | TRX3 (AT5G42980) | Pathway generated using literature input from pathway studio (Figure_5_SuppInfo). | Incorporated in manual pathway using input from pathway studio (Figure 10). |
| 1. H+ transporting ATPase | CaF1_JIE_07_E_03 | TC14586 | gb|ABN08957.1|  [Medicago truncatula] | AT3G28710 | No homology found in pathway studio. | Incorporated in manual pathway using input from published literature. (Figure 10). |
| 2**. CELLULAR TRANSPORT RELATED TRANSCRIPTS** |  |  |  |  |  |  |
| 1. **INTRACELLULAR TRANSPORTERS** 2. ABC transporter like protein | CaF1_WIE_19_A_07 | TC05017 | gb|ABE84066.1|  [Medicago truncatula] | AT2G34250 | No homology found in pathway studio | Incorporated in manual pathway using input from published literature. (Figure 10). |
| 1. Substrate transporter (carbohydrate) | CaF1_WIE_51_F_04 | TC16724 | gb|ABN08184.1|  [Medicago truncatula] | AT1G54730 | No homology found in pathway studio | Incorporated in manual pathway using input from published literature. (Figure 10). |
| 1. heavy metal transporter/ detoxyfying protein (FAR1 related sequence 6:FRS6) | CaF1_WIE_37_G_02 | TC07483 | gb|ABE93744.2|  [Medicago truncatula] | AT1G52520 | No homology found in pathway studio | Incorporated in manual pathway using input from published literature. (Figure 10). |
| 1. Translocase ( chloroplast 34) | CaF1_JIE_26_G_09 | TC14372 | sp|Q41009|TOC34_PEA | AT5G05000 | No homology found in pathway studio | Incorporated in manual pathway using input from published literature. (Figure 10). |
| 1. Polyol transporter protein | CaF1_JIE_27_E_08 | TC04362 | emb|CAJ29291.1|  [Lotus japonicus] | PLT5 (AT3G18830) | Pathway generated using literature input from pathway studio (Figure_6_SuppInfo). | Incorporated in manual pathway using input from pathway studio (Figure 10). |
| 1. **CELLULAR TRAFFICKING TRANSPORTERS** 2. Vacuolar sorting receptor | CaF1_WIE_45_A_0 | TC10023 | gb|AAF22842.1|AF209910_1  [Prunus dulcis] | VSR1 (AT3G52850) | Pathway generated using literature input from pathway studio (Figure_6_SuppInfo). | Incorporated in manual pathway using input from pathway studio (Figure 10). |
| 1. Clathrin coat assembly protein | CaF1_JIE_36_B_11 | TC17520 | ref|NP_194186.1|  [Arabidopsis thaliana | AT4G24550 | No homology found in pathway studio | Incorporated in manual pathway using input from published literature. (Figure 10). |
| 1. Secretory carrier membrane protein | CaF1_JIE_24_F_11 | TC15013 | ref|NP_179680.1|  [Arabidopsis thaliana | AT2G20840 | No homology found in pathway studio | Incorporated in manual pathway using input from published literature. (Figure 10). |
| 1. Nuclear pore complex protein | CaF1_WIE_48_B_10 | TC01108 | gb|ABE93939.2|  [Medicago truncatula] | AT5G51200 | No homology found in pathway studio | Incorporated in manual pathway using input from published literature. (Figure 10). |
| 1. Intrinsic protein of tonoplast | CaF1_WIE_34_E_11 | TC07483 | gb|ABE93744.2|  [Medicago truncatula] | TIP2 (AT3G26520) | Pathway generated using literature input from pathway studio (Figure_6_SuppInfo). | Incorporated in manual pathway using input from pathway studio (Figure 10). |
| 1. **INTRACELLULAR TRANSPORTATION SIGNAL GENERATORS** 2. TRK(A-N) signaling factor | CaF1_WIE_40_E_04 | TC04434 | gb|ABN05791.1|  [Medicago truncatula | AT4G00630 | No homology found in pathway studio. | Incorporated in manual pathway using input from published literature. (Figure 10). |
| 1. Type II B calcium ATPase | CaF1_WIE_09_A_05 | TC01484 | gb|AY147012.1|  [Medicago truncatula] | ACA2 (AT4G37640) | Pathway generated using literature input from pathway studio (Figure_6_SuppInfo). | Incorporated in manual pathway using input from pathway studio (Figure 10). |
| 1. **TRANSCRIPTION FACTOR RELATED TRANSCRIPTS** |  |  |  |  |  |  |
| 1. **TRANSCRIPTION FACTOR CONTAINING BASIC DOMAINS**   1. bZIP domain containing protein | CaF1_WIE_53_F_03 | TC16286 | gb|ABG90380.1|  [Caragana korshinskii] | AT5G42910 | No homology found in pathway studio. | Incorporated in manual pathway using input from published literature. (Figure 10). |
| 2. Homoeodomain leucine zipper like protein | CaF1_WIE_37_A_10 | TC05808 | gb|AAK84885.1|AF402604_1  [Phaseolus vulgaris] | REV (AT5G60690) | Pathway generated using literature input from pathway studio (Figure_7_SuppInfo). | Incorporated in manual pathway using input from pathway studio (Figure 10). |
| 3. MYB like transcription factor | CaF1_WIE_45_E_07 | TC09491 | gb|ABH02845.1|  [Glycine max] | MYB106 (AT3G01140) | Pathway generated using literature input from pathway studio (Figure_7_SuppInfo). | Incorporated in manual pathway using input from pathway studio (Figure 10). |
| 1. Helix loop helix domain containing transcription factor | CaF1_WIE_30_E_09 | TC15129 | gb|AAM63313.1|  [Arabidopsis thaliana] | AT1G05805 | No homology found in pathway studio. | Incorporated in manual pathway using input from published literature. (Figure 10). |
| 1. Zinc finger (CCHC type) | CaF1_JIE_03_B_09 | TC00757 | gb |XM_003529055|  [Glycine max] | AZF2 (AT3G19580) | Pathway generated using literature input from pathway studio (Figure_7_SuppInfo). | Incorporated in manual pathway using input from pathway studio (Figure 10). |
| 1. Heat shock family protein | CaF1_JIE_41_D_04 | TC04961 | emb|CAA87075.1|  [Glycine max] | HSF3 (AT5G16820) | Pathway generated using literature input from pathway studio (Figure_7_SuppInfo). | Incorporated in manual pathway using input from pathway studio (Figure 10). |
| 1. **TRANSCRIPTION FACTOR ASSOCIATORS** 2. Polynucleotidyl transferase (FAR1) | CaF1_JIE_03_C_03 | TC34562 | gb|ABD33394.2| | AT4G12850 | No homology found in pathway studio. | Incorporated in manual pathway using input from published literature. (Figure 10). |
| 1. Initiation factor 4a | CaF1_JIE_03_C_04 | TC02836 | gb|EDN17888.1|  [Botryotinia fuckeliana B05.10] | AT3G13920 | No homology found in pathway studio. | Incorporated in manual pathway using input from published literature. (Figure 10). |
| 1. Prefoldin (ILR3) | CaF1_WIE_34_F_11 | TC17935 | gb|ABE93168.1|  [Medicago truncatula] | AT5G54680 | No homology found in pathway studio. | Incorporated in manual pathway using input from published literature. (Figure 10). |
| 1. High mobility group B like protein | CaF1_WIE_18_H_03 | TC07452 | emb|CAJ38371.1|  [Plantago major] | HMGB3 (AT1G20696) | Pathway generated using literature input from pathway studio (Figure_7_SuppInfo). | Incorporated in manual pathway using input from pathway studio (Figure 10). |
| **4.SUGAR METABOLISM RELATED GENES** |  |  |  |  |  |  |
| 1. Sucrose synthase | GO935217 | TC11959 | gb|AF079851|  [Pisum sativum] | SUS4 ( AT3G43190) | Pathway generated using literature input from pathway studio (Figure_7_SuppInfo). | Incorporated in manual pathway using input from pathway studio (Figure 10). |
| 1. β Amylase | GO935221 | TC06159 | emb|CAI39244|  [Glycine max] | BAM1 (AT3G23920) | Pathway generated using literature input from pathway studio (Figure_7_SuppInfo). | Incorporated in manual pathway using input from pathway studio (Figure 10). |
| 1. Invertase | GO660552 | TC05218 | gb| NM111456|  [Arabidopsis thaliana] | AT3G05820 | No homology found in pathway studio. | Incorporated in manual pathway using input from published literature. (Figure 10). |
