# Supplementary material for: Fusarium oxysporum f.sp. ciceri Race 1 Induced Redox State Alterations Are Coupled to Downstream Defense Signaling in Root Tissues of Chickpea (Cicer arietinum L.)
Source: PLoS One. 2013 Sep 13;8(9):e73163. doi: 10.1371/journal.pone.0073163 (PMC3772884; doi:10.1371/journal.pone.0073163)
Supplement: Table S4 — Primer sequences with database references used for qRT PCR. (DOC) [file pone.0073163.s015.doc]

| **Supporting Table S4 Primer sequences with database references used for qRT PCR.** | | | |
| --- | --- | --- | --- |
| **Column1** | **Column2** | Column3 | **Column4** |
| **SL. NO.** | **PRIMER NAMES** |  | **PRIMER SEQUENCE** |
| **A.** | **CELLULAR REDOX RELATED ESTs** |  |  |
| 1 | PEROXIDASE (CaF1_JIE_03_E_09) | FORWARD | 5’ATG TAT GAG CAC CTG AGA GAG CA3’ |
|  |  | REVERSE | 5’CTA GCT AAT ATA AAT CTT CCA GCT CC3’ |
| 2 | RESPIRATORY BURST OXIDASE (CaF1_JIE_03_G_05) | FORWARD | 5’AGT GGT GAC CAA AGT GGT CTT CT3’ |
|  |  | REVERSE | 5’CTT CGT AGT CTT TAT AGT CTT GTG CTG3’ |
| 3 | H+ TRANSPORTING ATPase (CaF1_JIE_07_E_03) | FORWARD | 5’TTC AAA AGC TAG AAG GTC AGA CAT T3’ |
|  |  | REVERSE | 5’ATG AAC ATC GAA ATC ATG AGG AAC3’ |
| 4 | CATIONIC PEROXIDASE (CaF1_JIE_07_G_06) | FORWARD | 5’CTC TAG TTG GTA AGT CAT CAA CTT TTC3’ |
|  |  | REVERSE | 5’TGT CAA GAG CAC TAA AGT CCA ACC3’ |
| 5 | CYTOCHROME b561 FERRIC REDUCTASE (CaF1_WIE_18_D_06) | FORWARD | 5’AGG TAC TGG AAT ATT TAC CAC TAC CTT3’ |
|  |  | REVERSE | 5’AGT CCT TGT AAC GAT CTC CAA CAT3’ |
| 6 | GLUTATHIONE S TRANSFERASE (TAU 26) (CaF1_WIE_50_B_08) | FORWARD | 5’TAA CAA GAG CTT GGA GAT AAG CCA3’ |
|  |  | REVERSE | 5’CCA TAG GTC TCA TAA GTG TAA AAC CA3’ |
| 7 | CYTOCHROME B5 (CaF1_WIE_30_F_11) | FORWARD | 5’CTT GCT TTA ATA GTT AGT GTC AGG CC3’ |
|  |  | REVERSE | 5’TTC AGC TGC TCA CAA AGC CAT T3’ |
| 8 | NADP DEPENDENT OXIDOREDUCTASE (CaF1_WIE_33_E_02) | FORWARD | 5’TAC AGC TTG TTA CCT GCA GCT CA3’ |
|  |  | REVERSE | 5’TTC AAA GAA ACC AGC ATA AGC AGT3’ |
| 9 | NADH CYTOCHROME b5 REDUCTASE (CaF1_WIE_24_D_04) | FORWARD | 5’ATC ACT CCA ATG TTC CAA GTT GC3’ |
|  |  | REVERSE | 5’TAG TTG GTA GCA AGA CCA TCA AGT T3’ |
| 10 | F-type THIOREDOXIN (CaF1_WIE_04_C_09) | FORWARD | 5’TGA TAA GAC CGT CGT CCT CGA3’ |
|  |  | REVERSE | 5’ATC TTG GTT GCA ATC AAG ATT TA3’ |
| 11 | 20G-Fe(II) OXYGENASE (CaF1_JIE_25_A_06) | FORWARD | 5’AAA GTT TCT ACC AAT CTG AGA TAG TAA AG3’ |
|  |  | REVERSE | 5’ATG CCA AAT GAA GAA TGC ACT TTA C3’ |
| 12 | Fe SUPEROXIDE DISMUTASE (CaF1_WIE_30_B_01) | FORWARD | 5’AGA ACA TGG TTC CCC TGA GGA3’ |
|  |  | REVERSE | 5’ ATG GAG TTT GGT CCA GTG AGA GG3’ |
| 13 | QUINONE OXIDOREDUCTASE (CaF1_WIE_38_G_09) | FORWARD | 5’ACA CCA CTT CCA AAA CAT GGA TC3’ |
|  |  | REVERSE | 5’AGT GAA TCC TCA AAT ACA CTG TTA TTG3’ |
|  |  |  |  |
| **B.** | **ESTs RELATED TO CELLULAR TRANSPORT** |  |  |
| 1 | ABC TRANSPORTER LIKE PROTEIN (CaF1_WIE_19_A_07) | FORWARD | 5’TGG AAG GAA TCT GAA TAT GGA GGT3’ |
|  |  | REVERSE | 5’AGA ACA GTG CAT GGA AAG GAT TG3’ |
| 2 | SUBSTRATE TRANSPORTER (CARBOHYDRATE) (CaF1_WIE_51_F_04) | FORWARD | 5’AAG AAT GCT GAT GTT TAT AAA GAG GC3’ |
|  |  | REVERSE | 5’ATT TGA AAT AAG CCA ATA ATG CTG G3’ |
| 3 | VACUOLAR SORTING RECEPTOR (CaF1_WIE_45_A_0) | FORWARD | 5’TTC AGA ATC CAT ATA TGA CCT TAT TCT3’ |
|  |  | REVERSE | 5’AAC TGC CAG TCA AGA AGG AAA GT3’ |
| 4 | TRK(A-N) SIGNALING FACTOR (CaF1_WIE_40_E_04) | FORWARD | 5’ACC TAG TCT TCA ACT AGC AGC TGC T3’ |
|  |  | REVERSE | 5’TAA GCT CAG CTA GAT GTC GGC A3’ |
| 5 | CLATHRIN COAT ASSEMBLY PROTEIN (CaF1_JIE_36_B_11) | FORWARD | 5’TGC GAG CTG AAT TCA ACT CAA3’ |
|  |  | REVERSE | 5’AGT TGT GTG TCC AAC TGC TCC A3’ |
| 6 | TYPE II-B CALCIUM ATPase (CaF1_WIE_09_A_05) | FORWARD | 5’TTC CTT GGA GTC CTT GGC AT3’ |
|  |  | REVERSE | 5’AAT TAA CAT TGT TCC GGT CTT CC3’ |
| 7 | SECRETORY CARRIER MEMBRANE PROTEIN (CaF1_JIE_24_F_11) | FORWARD | 5’TCC TAC CAT CTG GTT TCT TGC TAT3’ |
|  |  | REVERSE | 5’AGC CAA ACT TCA GAG CAC TGT CC3’ |
| 8 | POLYOL TRANSPORTER (CaF1_JIE_27_E_08) | FORWARD | 5’AAC CGT TTT ATC TTA TGT TGC AAC T3’ |
|  |  | REVERSE | 5’CCT ATT CAC CAC AAC TCC CAT AGC3’ |
| 9 | TRANSLOCASE (CHLOROPLAST 34) (CaF1_JIE_26_G_09) | FORWARD | 5’TTC CTA TCG CCG CTC TCT CTT3’ |
|  |  | REVERSE | 5’AAG TTC TAG CAA CTT GGT CTG CG3’ |
| 10 | NUCLEAR PORE COMPLEX PROTEIN (CaF1_WIE_48_B_10) | FORWARD | 5’CAA TAA AAA TCT TGA GGA GCA AGC3’ |
|  |  | REVERSE | 5’TTT CAG ATC GAT CTT CAA GCA TT3’ |
| 11 | INTRINSIC PROTEIN OF TONOPLAST (CaF1_WIE_34_E_11) | FORWARD | 5’TTC ATG GAT GCT CCG GTG A3’ |
|  |  | REVERSE | 5’TGA CCC AAA GAA GGG TAA TAT TGG3’ |
| 12 | HEAVY METAL TRANSPORTER / DETOXIFYING PROTEIN (FRS6) (CaF1_WIE_37_G_02) | FORWARD | 5’ATG TCT GCT ACA CAC CAT GGT GA3’ |
|  |  | REVERSE | 5’CTT CCT TTA ACT TCT TGT GAA GAG C3’ |
|  |  |  |  |
| **C.** | **TRANSCRIPTION FACTOR RELATED ESTs** |  |  |
| 1 | ZINC FINGER (CCHC TYPE) (CaF1_JIE_03_B_09) | FORWARD | 5’ CAT GCA TCA GTT GGA TGT AAT GTC 3’ |
|  |  | REVERSE | 5’ TCA GCC TAT ACA CAT TGT CTT CTT TC 3’ |
| 2 | POLYNUCLEOTIDYL TRANSFERASE (FAR1) (CaF1_JIE_03_C_03) | FORWARD | 5’ TTG TCT CAA GAT TTG TAA AGG ATC AC 3’ |
|  |  | REVERSE | 5’ AGT TCA TCT TGG CGC TCC AA 3’ |
| 3 | INITIATION FACTOR 4a (CaF1_JIE_03_C_04) | FORWARD | 5’ TGC TGT AGA ACT GCT CAA TCT CAC 3 |
|  |  | REVERSE | 5’ ACC GAA TTG GTC GTG GTG GT 3’ |
| 4 | bZIP DOMAIN CONTAINING PROTEIN (CaF1_WIE_53_F_03) | FORWARD | 5’ CTA ATG GTG GTG ATA CTT CTT CTG TG 3’ |
|  |  | REVERSE | 5’ CTA TTC TTT ATC ATT CTT CTC TGC CTC 3’ |
| 5 | HOMOEODOMAIN LEUCINE ZIPPER LIKE PROTEIN (CaF1_WIE_37_A_10) | FORWARD | 5’ CAA ACA ATT GGA AAG AGA TTA TGG T 3’ |
|  |  | REVERSE | 5’ TTC TTG ATT TCA ATT CCT TAA CCT C 3’ |
| 6 | MYB LIKE TRANSCRIPTION FACTOR (CaF1_WIE_45_E_07) | FORWARD | 5’ ACT GCG GTT GGC TTC AAG AT 3’ |
|  |  | REVERSE | 5’ CCA GTA GTA TAT ATC CGG CAT ACT ATT 3’ |
| 7 | HEAT SHOCK FAMILY PROTEIN (CaF1_JIE_41_D_04) | FORWARD | 5’ TCA TCG AAC TCG TCG CCG T 3’ |
|  |  | REVERSE | 5’ TGC AAA GCG ATT TCA TTG CA 3’ |
| 8 | HELIX-LOOP-HELIX DOMAIN CONTAINING TRANSCRIPTION FACTOR (CaF1_WIE_30_E_09) | FORWARD | 5’ TTA TTC AGA CAT GCT GGA TTT GG 3’ |
|  |  | REVERSE | 5’ ATT ATG TGC TAG GTT TGC ATC CA 3’ |
| 9 | PREFOLDIN (ILR3) (CaF1_WIE_34_F_11) | FORWARD | 5’ TAG ATC GGA TTC ATG TGC TCC T 3’ |
|  |  | REVERSE | 5’ CAA GAT TGA GCC TAA TTC AGC AA 3’ |
| 10 | HIGH MOBILITY GROUP B LIKE PROTEIN (CaF1_WIE_18_H_03) | FORWARD | 5’ AAG ACC TCC GAG CGC TTT CT 3’ |
|  |  | REVERSE | 5’ TTC CAT TTC TCT CCT CCA GCC 3’ |
| **D.** | **SUGAR METABOLISING ESTs** |  |  |
|  |  |  |  |
| 1 | β AMYLASE (GO935221) | FORWARD | 5' GCT ATT GGT AAA TAA CCA TC 3' |
|  |  | REVERSE | 5' CTA GTG ATT GAT GAG TCC TGA 3' |
| 2 | INVERTASE (GO660552) | FORWARD | 5' GAG ATC CAA GCT TTG TTC 3' |
|  |  | REVERSE | 5' GAG TAA CAT CTA CTG ATC 3' |
| 3 | SUCROSE SYNTHASE (GO935217) | FORWARD | 5' GGA GAT CAG CGA CGC GTT CG 3' |
|  |  | REVERSE | 5' GAG TCC TGA GTA ACT GGT 3' |
| **E.** | **CALLOSE DEGRADING β 1-3 ENDO GLUCANASE (Q9XFW9_CICAR)** | FORWARD | 5' AAC AAT ATA CCA CCA GCA A 3' |
|  |  | REVERSE | 5' CAT TGT TTG TGG CTA GAC TT 3' |
