# Supplementary material for: Fusarium oxysporum f.sp. ciceri Race 1 Induced Redox State Alterations Are Coupled to Downstream Defense Signaling in Root Tissues of Chickpea (Cicer arietinum L.)
Source: PLoS One. 2013 Sep 13;8(9):e73163. doi: 10.1371/journal.pone.0073163 (PMC3772884; doi:10.1371/journal.pone.0073163)
Supplement: Figure S9 — Network showing interaction between sugar metabolizers and redox regulators. SUS4, sucrose synthase 4; BAM1, beta amylase; CDKB1.1, serine threonine kinase; TUF, vacuolar ATPase. (PDF) [file pone.0073163.s009.pdf]

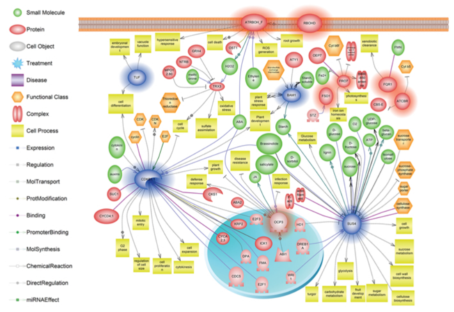

Small Molecule
Protein
Cell Object
Treatment
Disease
Functional Class
Complex
Cell Process
Expression
Regulation
MolTransport
ProtModification
Binding
PromoterBinding
MolSynthesis
ChemicalReaction
DirectRegulation
miRNAEffect
ATRBOH_F
RBOHD
embryonal developmen t
vacuole function
hypersensitive response
cell death
OST1
ROS generation
root growth
GRX4
NTRB
MPO1 2.80
methi onine
H2O2
TRX3
TUF
Ethylen e
ATY1
BAM1
Starch, soluble
plant stress response
Plant developmen t
Thioredoxi n reductase
oxidative stress
cell differentiation
CDK
CDK N
cyclin
E2F
cell cycle
sulfate assimilation
ABA
Starch
Cyt b6f
OEP7
Fe2+
FRO7
FSD1
STZ
photosynthesi s
iron ion homeostasis
xenobiotic clearance
FMN
Cyt b5
FQR1
CB5-E
ATCBR
O2
UDP-glucose
D-glucose
beta-glucan
ATP
D-fructose
lignin
sucrose
Isomalt ulose
Glucose metabolism
sucrose transporte r
sucrose-phosphate synthase
sugar porter
cellulose synthase
cytokini n
auxins
CDKB1;1
SUC1
CYCD4;1
Brassinolide
salicylate
D-sorbitol
disease resistance
plant growth
defense response
JA
infection response
CKS1
ABA2
E2F3
KRP2
OCP3
HD1
CYCA 2;3
ICK1
DPA
FMA
ABI1
DREB1 A
CDC5
E2F1
WRI 1
SUS4
mitotic entry
G2 phase
regulation of cell size
cell proliferatio n
cytokinesis
cell expansion
glycolysis
turgor
carbohydrate metabolism
fruit develop ment
sugar metabolism
cell growth
sucrose metabolism
cell wall biosynthesis
cellulose biosynthesis
